# Broad-Host-Range Expression Reveals Native and Host Regulatory Elements That Influence Heterologous Antibiotic Production in Gram-Negative Bacteria

**DOI:** 10.1128/mBio.01291-17

**Published:** 2017-09-05

**Authors:** Jia Jia Zhang, Xiaoyu Tang, Michelle Zhang, Darlene Nguyen, Bradley S. Moore

**Affiliations:** a Center for Marine Biotechnology and Biomedicine, Scripps Institution of Oceanography, University of California at San Diego, La Jolla, California, USA; b Genomic Medicine, J. Craig Venter Institute, La Jolla, California, USA; c Skaggs School of Pharmacy and Pharmaceutical Sciences, University of California at San Diego, La Jolla, California, USA; University of British Columbia

**Keywords:** Gram negative, heterologous expression, *Proteobacteria*, broad-host-range vector, violacein

## Abstract

Heterologous expression has become a powerful tool for studying microbial biosynthetic gene clusters (BGCs). Here, we extend the transformation-associated recombination cloning and heterologous expression platform for microbial BGCs to include Gram-negative proteobacterial expression hosts. Using a broad-host-range expression platform, we test the implicit assumption that biosynthetic pathways are more successfully expressed in more closely related heterologous hosts. Cloning and expression of the violacein BGC from *Pseudoalteromonas luteoviolacea* 2ta16 revealed robust production in two proteobacterial hosts, *Pseudomonas putida* KT2440 and *Agrobacterium tumefaciens* LBA4404, but very little production of the antibiotic in various laboratory strains of *Escherichia coli*, despite their closer phylogenetic relationship. We identified a nonclustered LuxR-type quorum-sensing receptor from *P. luteoviolacea* 2ta16, PviR, that increases pathway transcription and violacein production in *E. coli* by ∼60-fold independently of acyl-homoserine lactone autoinducers. Although *E. coli* harbors the most similar homolog of PviR identified from all of the hosts tested, overexpression of various *E. coli* transcription factors did not result in a statistically significant increase in violacein production, while overexpression of two *A. tumefaciens* PviR homologs significantly increased production. Thus, this work not only introduces a new genetic platform for the heterologous expression of microbial BGCs, it also challenges the assumption that host phylogeny is an accurate predictor of host compatibility.

## IMPORTANCE

Although Gram-positive heterologous hosts such as *Streptomyces* have been developed and optimized to support diverse secondary metabolic reactions, there has been comparatively less work on Gram-negative hosts, some of which grow faster and are easier to work with. This work presents a new genetic platform for direct cloning and broad-host-range heterologous expression of BGCs in Gram-negative proteobacterial expression hosts, and we leverage this platform to uncover regulatory elements that influence violacein expression from *Pseudoalteromonas*. Although it is often assumed that BGCs will be more successfully expressed in more closely related hosts, our work suggests that this may not be a general rule of thumb, as heterologous production of natural products can be influenced by specific host regulatory and/or biosynthetic elements, and the identity and effectiveness of those elements are difficult to predict. We argue for the use of a diverse set of heterologous hosts, which may also provide insights into BGC mechanism and function.

## INTRODUCTION

Biosynthetic gene clusters (BGCs) for the production of specialized metabolites can occupy up to 10% of a bacterial genome (1), and DNA sequencing and new bioinformatics tools continue to uncover previously undetected biosynthetic potential (2). The metabolic cost of evolving, gaining, or maintaining a BGC is high, as some can exceed 100 kb in size and encode dozens of proteins that catalyze synchronized enzymatic reactions. Despite their prominence, most BGCs have not been characterized (3), and while the pharmacology of many natural products has been studied in animal or cancer cell lines, their biological role within their native context is often not understood. Thus, many questions regarding microbial BGCs remain to be explored.

Heterologous expression has become a powerful tool for the investigation of microbial BGCs for several reasons. First, BGCs have become easier to clone. Techniques that rely upon homologous recombination in yeast or *Escherichia coli* have greatly simplified the process of cloning large fragments of DNA (4, 5), and in the near future, many BGCs will likely be obtained directly from DNA synthesis. Second, heterologous expression of a defined set of genes allows for direct connection of BGCs to their small-molecule products and simultaneously establishes a boundary of essential elements. Third, utilizing a cloning and heterologous expression platform eliminates the need to develop new genetic tools for the manipulation of each new genus or species of interest. Instead, a set of universally applicable tools can be optimized and applied to BGCs from any source. Thus, targeted changes can readily be made to “cryptic” or “silent” BGCs that do not produce small molecules under normal laboratory culture conditions (3). Despite these advantages, successful heterologous production of small molecules from new BGCs remains a major bottleneck (6), especially for pathways that may utilize uncharacterized and/or nonclustered regulatory or biosynthetic elements.

One important consideration is the choice of an expression host. Despite numerous reports of successful heterologous expression of BGCs from various sources, researchers have historically relied upon a rather limited set of hosts, focusing heavily on the development and optimization of high-GC-content Gram-positive organisms such as *Streptomyces*, including genetically minimized strains of *Streptomyces coelicolor* and *Streptomyces avermitilis* (7, 8). These strains have been specifically engineered to optimize secondary metabolite production through deletion of native pathways to remove sinks for carbon and nitrogen and introduction of specific mutations in transcriptional and translational machinery that have been empirically shown to increase secondary metabolite production. Far less attention has been paid to Gram-negative hosts, despite growing interest in Gram-negative bacteria as sources of new antibiotics (9). Gram-negative hosts that have been successfully utilized for the heterologous expression of BGCs include *Myxococcus xanthus*, *Pseudomonas putida*, and *E. coli* (10). Although they have not been leveraged or optimized to the same extent as *Streptomyces* hosts, some of these organisms grow faster and are easier to work with.

A general assumption often stated or implied is that the best heterologous host will likely be an organism most similar or closely related to the original source of the BGC. Although theoretically appealing, this hypothesis has not been rigorously tested and there is limited empirical evidence to support this claim. Müller and coworkers hypothesized that the tubulysin gene cluster from *Cystobacter* sp. strain SBCb004 would be better expressed in *M. xanthus* than in *P. putida* because of the closer relationship between the two *Deltaproteobacteria*, and indeed, the authors observed 100-fold higher production in *M. xanthus* than in *P. putida* (11). However, production titers from *M. xanthus* were still lower than those from the native producer, and reasons for the observed differences in production level were not investigated. One possible rationale is that production of a natural product could involve chaperones or machinery not directly encoded within a gene cluster. Consequently, heterologous expression of BGCs often necessitates that host factors complement any missing activities. This leads to the impression that more closely related organisms will be more compatible heterologous hosts. However, whether this is generally the case remains to be established.

Previously, we and others constructed shuttle vectors that combine the capabilities of transformation-associated recombination (TAR) cloning in yeast, maintenance and genetic manipulation in *E. coli*, and heterologous expression in *Streptomyces* hosts (12–14). Recently, we improved the efficiency of our cloning method and expanded our platform to include *Bacillus* hosts (15, 16). We have used this platform to successfully interrogate the biosynthesis and bioactivity of several natural-product BGCs (17–19). In this work, we extend the platform to include Gram-negative proteobacterial hosts, constructing a direct cloning and broad-host-range expression vector and validating three hosts: the alphaproteobacterium *Agrobacterium tumefaciens* LBA4404 and the *Gammaproteobacteria P. putida* KT2440 and *E. coli*. Using the BGC for the antibacterial pigment violacein from *Pseudoalteromonas luteoviolacea* 2ta16, which also belongs to the class *Gammaproteobacteria*, we (i) validate the extension of our genetic platform, (ii) test the assumption that more closely related hosts perform better, and (iii) investigate specific native and host factors that control violacein expression. Our results suggest that investigation of microbial BGCs will be aided by the development and use of diverse heterologous hosts, as the identity and activity of host elements that influence heterologous antibiotic production are difficult to predict.

## RESULTS AND DISCUSSION

### Construction of a direct cloning, broad-host-range expression vector for proteobacterial hosts.

**FIG 1:**
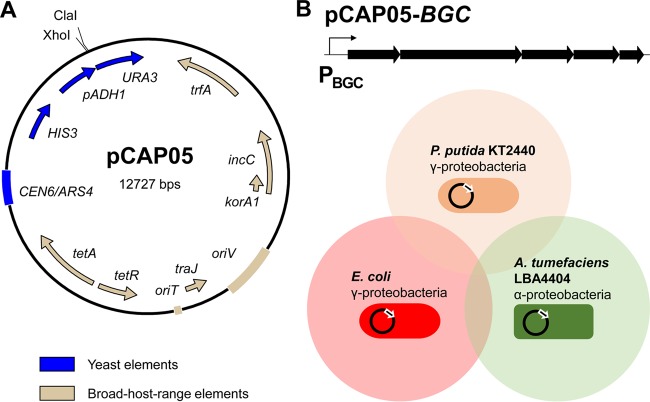
Direct cloning and proteobacterial broad-host-range heterologous expression of microbial BGCs. (A) Organization of TAR cloning and broad-host-range expression vector pCAP05 composed of elements for maintenance (*CEN6*/*ARS4*), selection (*HIS3*), and counterselection (*pAHD1*, *URA3*) in yeast (blue) and broad-host-range maintenance (*oriV*, *trfA*) and selection (*tetA*, *tetR*) in Gram-negative bacteria (tan). Additionally, *incC* and *korA1* regulate plasmid maintenance and *oriT* and *traJ* enable conjugal transfer of pCAP05. The vector can be linearized for cloning by digestion with ClaI and XhoI. (B) Schematic illustration of the broad-host-range heterologous expression concept. A generic microbial BGC cloned into pCAP05 is depicted above three proteobacterial expression hosts that were selected and validated in this study.

A new TAR cloning vector, pCAP05, was constructed by combining yeast elements for cloning with Gram-negative broad-host-range elements for heterologous expression (Fig. 1A). Broad-host-range elements originate from plasmid pRK442(H), a broad-host-range vector of the RK2 family. The RK2 replicon and its derivatives, which belong to the IncP incompatibility group, have been shown to replicate at low copy number (<30 in *E. coli*) in a wide range of Gram-negative bacteria through the *oriV* origin of replication and the essential *trfA* gene, which controls plasmid copy number and host range (20). Notably, however, RK2 replicons do not propagate in *Bacteroides* or *Myxococcus* (21). RK2 derivative plasmids have previously been utilized as versatile tools for broad-host-range screening of metagenomic DNA libraries (22, 23). Given the abundance of publicly available DNA sequence information and advances in bioinformatic detection of BGCs, direct cloning of targeted pathways has become an attractive alternative to untargeted library generation and screening (3). Thus, pCAP05 includes elements for maintenance, selection, and counterselection in *Saccharomyces cerevisiae* to leverage an improved TAR cloning method we described previously (15), which allows for the use of short capture arm sequences and improves cloning efficiency.

We assembled pCAP05 using Gibson Assembly and validated that the empty vector could be introduced into *S. cerevisiae* VL6-48N and selected against or for in histidine-deficient medium with or without 5-fluoroorotic acid (5-FOA), respectively (see Fig. S1A to D in the supplemental material). *HIS3* was chosen as the yeast selective marker to make pCAP05 orthogonal to our original series of capture vectors, which use TRP1 and are designed for the heterologous expression of BGCs in *Streptomyces* hosts (13, 15). We then validated that pCAP05 could be transferred to two proteobacterial expression hosts beyond *E. coli*, *P. putida* KT2440 and *A. tumefaciens* LBA4404, and selected for with tetracycline (Fig. S1E and F). *P. putida* KT2440 is a metabolically versatile and biosafety-certified host that has been successfully utilized for the heterologous expression of several BGCs (24). Its genome has been sequenced, and it is amenable to genetic manipulation. Further, KT2440 was shown to possess a promiscuous phosphopantetheinyl transferase capable of posttranslationally activating carrier proteins involved in natural-product biosynthesis from myxobacteria and *Streptomyces*, which is an essential host trait for the heterologous expression of carrier protein-utilizing BGCs (25). *A. tumefaciens* LBA4404 is a biotechnological strain commonly used for plant transformation. LBA4404 was constructed from its immediate precursor, *A. tumefaciens* LBA4213, by the deletion of a large region of the tumor-inducing (Ti) plasmid, rendering it avirulent (26). Taken together with various laboratory strains of *E. coli*, these hosts represent phylogenetically diverse *Proteobacteria* that are compatible with pCAP05 for broad-host-range heterologous expression testing of microbial BGCs (Fig. 1B).

10.1128/mBio.01291-17.1 FIG S1  Construction and validation of pCAP05. (A) Approach used to combine elements from pRK442(H) and pARS-VN using Gibson Assembly. Elements maintained are shown in bold type. (B) Confirmation by restriction digestion with NdeI. (C) Transfer to *S. cerevisiae* VL6-48N and plating on selective medium without histidine and with 5-FOA. (D) Transfer to *S. cerevisiae* VL6-48N and plating on selective medium without histidine. (E) Transfer to *P. putida* KT2440 by electroporation with selection with tetracycline at 15 μg/ml in LB medium. (F) Transfer to *A. tumefaciens* LBA4404 by electroporation with selection with tetracycline at 5 μg/ml in YM medium. Download FIG S1, PDF file, 0.3 MB. Copyright © 2017 Zhang et al. 2017 Zhang et al. This content is distributed under the terms of the Creative Commons Attribution 4.0 International license.

### Cloning and heterologous expression of *vio2ta16*.

To validate our broad-host-range heterologous expression platform, we targeted the violacein BGC (*vio2ta16*) from the marine bacterium *P. luteoviolacea* 2ta16. *vio2ta16* was selected for several reasons. The BGC is compact in size (∼8 kb), possesses a simple single-operon organization, and produces an easily detectable purple pigment. Violacein is produced by a range of *Betaproteobacteria* and *Gammaproteobacteria*, including *P. luteoviolacea*, but the violacein BGC is not broadly conserved across all members of the genus *Pseudoalteromonas*. We efficiently TAR cloned *vio2ta16* into pCAP05 in yeast and transferred the construct to *E. coli* for verification by restriction digestion (Fig. S2A and B).

10.1128/mBio.01291-17.2 FIG S2  Validation of pCAP05-*vio2ta16* and pET28a-*vio2ta16*. (A) DNA used for TAR cloning. Three fragments was amplified from *P. luteoviolacea* 2ta16 gDNA and combined with pCAP05 digested with ClaI and XhoI. A 0.5-µg sample of each DNA fragment was transferred to *S. cerevisiae* VL6-48N spheroplasts and plated on selective medium without histidine and with 5-FOA. (B) Vector map of pCAP05-*vio2ta16* along with digestion confirmation from independent clones. (C) Expected restriction digestion fragments of pET28a-*vio2ta16* obtained with DraI, EcoRV, and NcoI are listed in a table, where bands that could be visualized in the gel are in bold. The construct was also sequenced at the insertion site with the T7 and T7 terminator primers (data not shown). Download FIG S2, PDF file, 0.3 MB. Copyright © 2017 Zhang et al. 2017 Zhang et al. This content is distributed under the terms of the Creative Commons Attribution 4.0 International license.

Within the phylum *Proteobacteria*, *A. tumefaciens* belongs to the taxonomic class *Alphaproteobacteria*, while *P. luteoviolacea*, *E. coli*, and *P. putida* all belong to *Gammaproteobacteria*. Telescoping multiprotein phylogenetic analysis of 104 gammaproteobacterial genomes resulted in a well-resolved phylogenetic tree that places the taxonomic orders to which *P. luteoviolacea* and *E. coli* belong (*Alteromonadales* and *Enterobacteriales*, respectively) within the same node, while the order of *P. putida* (*Pseudomonadales*) falls within a sister clade (27). Thus, broad-host-range expression of *vio2ta16* with the selected hosts allowed us to probe how host relationships across taxonomic order and class, as well as across different strains of *E. coli*, affect heterologous antibiotic production.

**FIG 2:**
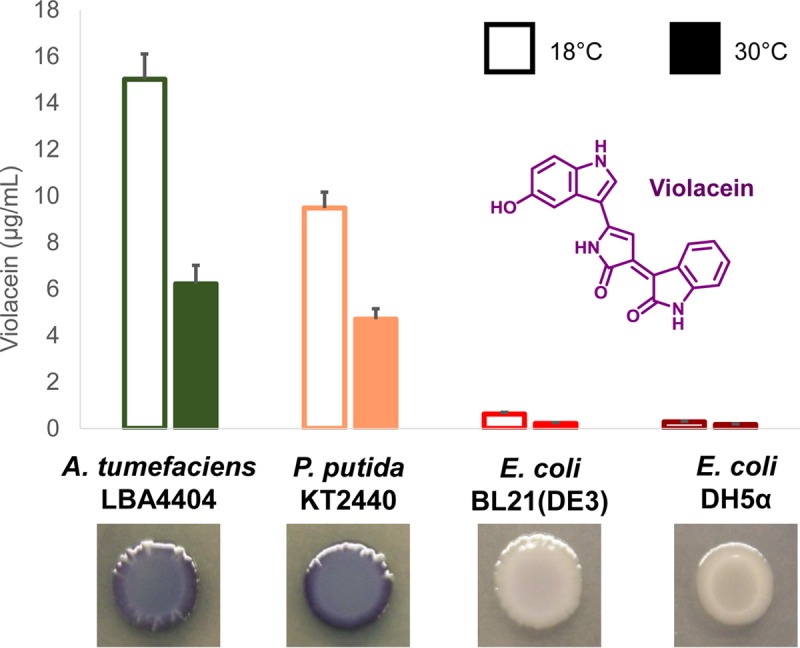
Heterologous expression of *vio2ta16* challenges the assumption that more closely related hosts perform better. Heterologous violacein production by four proteobacterial host strains was determined by extraction and HPLC quantification after 48 h of liquid culture in LB medium at 18 or 30°C. The data plotted are the mean ± standard error from two independent experiments (total *n* = 6). Plate images were taken after 48 h of growth at 30°C.

Despite the close phylogenetic relationship between *P. luteoviolacea* and *E. coli*, violacein was produced at very low levels by various laboratory strains of *E. coli*, including DH5α and BL21(DE3). Other *E. coli* strains, including Top 10, DH10B, BL21-Gold(DE3), Rosetta(DE3), and BW25113, were tested with very similar results (data not shown); thus, DH5α and BL21(DE3) were selected as two representative *E. coli* strains moving forward. Violacein production was not detectable by eye on any plates incubated at 37°C. However, we observed subtle differences in production that grew more pronounced over time at lower temperatures. At or below 30°C, low levels of violacein production could be visualized on plates of BL21(DE3) but not DH5α. Extraction of liquid cultures and quantification by high-performance liquid chromatography (HPLC) confirmed that BL21(DE3) was capable of producing approximately twice as much violacein as DH5α at 18°C (0.63 ± 0.07 μg/ml versus 0.28 ± 0.04 μg/ml), consistent with plate observations (Fig. 2). These observations indicated that even very minor changes in the host genotype at the strain level can result in fold changes in heterologous compound production.

Conversely, violacein production was readily detectable on plates of *P. putida* and *A. tumefaciens* transformed with pCAP05-*vio2ta16* and incubated at 30°C, the preferred growth temperature for these organisms. Extraction from *P. putida* liquid cultures and quantification confirmed the robust production of just under 10 μg of violacein/ml of culture after 48 h at 18°C (Fig. 2). Interestingly, the highest levels of production were obtained with *A. tumefaciens*, the least related host tested, which produced approximately 50% more violacein than *P. putida* at 18°C (Fig. 2). Production by all of the hosts was higher at 18°C than 30°C. Violacein production yield was not normalized to cell density, however, as the compound absorbs strongly at 600 nm and the *A. tumefaciens* heterologous host does not grow homogeneously in LB medium. Differences in growth or metabolism at different temperatures or in different host strains certainly affects heterologous production titers. Furthermore, the absolute plasmid copy number of pCAP05-*vio2ta16* in the hosts tested under the given cultivation conditions is not known either. Thus, our titer comparisons are only qualitative and not rigorously quantitative.

We also characterized violacein production levels from the native producer, *P. luteoviolacea* 2ta16, for qualitative comparison. Although production was relatively stable at 30°C (13.52 ± 1.36 μg/ml), it was highly inconsistent at 18°C (22.49 ± 11.77 μg/ml, Fig. S3). The reason for the spread at low temperature was not determined. Regardless, *P. luteoviolacea* 2ta16 is capable, at both growth temperatures, of producing more violacein than all of the heterologous hosts tested.

10.1128/mBio.01291-17.3 FIG S3  Quantification of violacein production by *P. luteoviolacea* 2ta16 grown at two different temperatures. The data from three independent experiments are in black, blue, and red. Download FIG S3, PDF file, 0.1 MB. Copyright © 2017 Zhang et al. 2017 Zhang et al. This content is distributed under the terms of the Creative Commons Attribution 4.0 International license.

**FIG 3:**
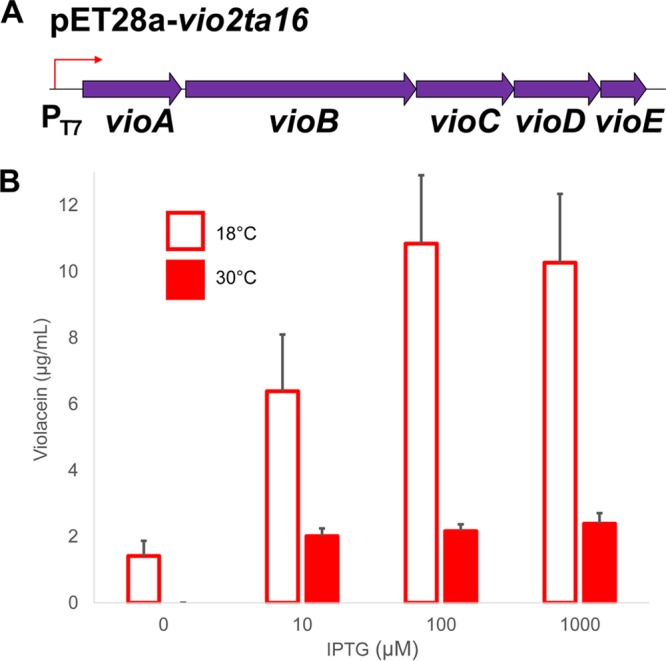
The *vio2ta16* native promoter is not efficiently activated in *E. coli*. (A) Schematic illustration of the pET28a-*vio2ta16* construct. The native *vio2ta16* promoter was replaced by transfer of the biosynthetic operon into expression vector pET28a, placing the pathway under the direct control of the T7 promoter. (B) Heterologous violacein production from *E. coli* BL21(DE3) harboring pET28a-*vio2ta16*. Cultures were grown to an OD_600_ of 0.5 to 0.6 at 37°C before induction with different concentrations of IPTG as shown. Following induction, liquid cultures were incubated for 24 h with shaking at 18 or 30°C before extraction and violacein quantification by HPLC. The data plotted are the mean ± standard error from two independent experiments (total *n* = 6).

It was previously reported that the violacein BGC from *Pseudoalteromonas* sp. strain 520P1 could not be expressed from its native promoter in *E. coli* (28). To test whether promoter recognition was also responsible for low violacein production from *vio2ta16*, we replaced the native pathway promoter by λ Red recombination-mediated PCR targeting (29), moving the biosynthetic operon into pET28a and placing it under the direct control of the T7 promoter (Fig. 3A and S2C). Promoter replacement revealed that strain BL21(DE3) is indeed capable of supporting high levels of violacein production, up to approximately 11 μg/ml (Fig. 3B), strongly suggesting that the *vio2ta16* promoter is only active at low levels in *E. coli* hosts and that this is the primary reason for the low production level observed. Again, production levels were higher at 18°C than at 30°C, indicating that temperature plays an important role in heterologous violacein production and suggesting that the violacein biosynthetic enzymes are more stable or correctly folded at a lower temperature.

### Regulation of the *vio2ta16* promoter.

To gain a more detailed understanding of why the native *vio2ta16* promoter was well recognized in *P. putida* and *A. tumefaciens* but not in *E. coli*, we set out to identify specific regulatory elements involved in *vio2ta16* regulation. Although the violacein BGC from many *Betaproteobacteria* and *Gammaproteobacteria* has been identified, a complete regulatory circuit controlling violacein expression has been fully characterized in only one bacterial species, *Chromobacterium violaceum*. In *C. violaceum*, violacein production is controlled by CviR, a LuxR-type quorum-sensing receptor that binds to and activates the transcription of the violacein promoter in the presence of its cognate autoinducer, an acyl-homoserine lactone (AHL) produced by CviI (30). Like the *luxI*-*luxR* quorum-sensing signal receptor pair from *Vibrio fischeri*, *cviI* and *cviR* are adjacent within the genome. It is notable that although the CviI-CviR pair has been confirmed to regulate violacein production in two strains of *C. violaceum* (ATCC 31532 and ATCC 12472), *C. violaceum* ATCC 31532 produces a C_6_ homoserine lactone (C_6_-HSL) and is inhibited by long-chain HSLs (C_10_ to C_14_), while *C. violaceum* ATCC 12472 produces 3-hydroxy-C_10_-HSL and is inhibited by short-chain HSLs (C_4_ to C_8_) (31, 32). Thus, the two circuits are functional opposites of one another.

We queried the genome of *P. luteoviolacea* 2ta16 with DELTA-BLAST (33) for CviR homologs that could be involved in violacein regulation. We identified seven candidates, which we named PLR1 to PLR7, four of which appear to be canonical LuxR-type receptors and three of which appear to be response regulators in a two-component regulatory system, as evidenced by the presence of N-terminal phosphorylation sites (Fig. S4). Conserved domains for AHL autoinducer binding were not identified in any of the seven candidate receptors by the NCBI conserved-domain identifier (34). Of six amino acid residues important for AHL binding (35, 36), three or fewer residues are conserved in any single candidate receptor identified (Fig. S5). Furthermore, *P. luteoviolacea* 2ta16 does not possess any homologs of the AHL autoinducer synthase CviI or LuxI.

10.1128/mBio.01291-17.4 FIG S4  Results of a DELTA-BLAST search querying the genome of *P. luteoviolacea* 2ta16 by using CviR from *C. violaceum* ATCC 31532. All of the hits possess E values above the significance threshold. The top panel shows sequence alignments. The table lists candidates with putative two-component system response regulators in blue. Download FIG S4, PDF file, 0.2 MB. Copyright © 2017 Zhang et al. 2017 Zhang et al. This content is distributed under the terms of the Creative Commons Attribution 4.0 International license.

10.1128/mBio.01291-17.5 FIG S5  Multiple-sequence alignment of CviR, LuxR, and PLR1-7 generated with CLUSTAL O(1.2.4). Amino acid residues conserved across quorum-sensing signal receptors within the autoinducer- and DNA-binding domains are highlighted in yellow and gray, respectively. Download FIG S5, PDF file, 0.1 MB. Copyright © 2017 Zhang et al. 2017 Zhang et al. This content is distributed under the terms of the Creative Commons Attribution 4.0 International license.

**FIG 4:**
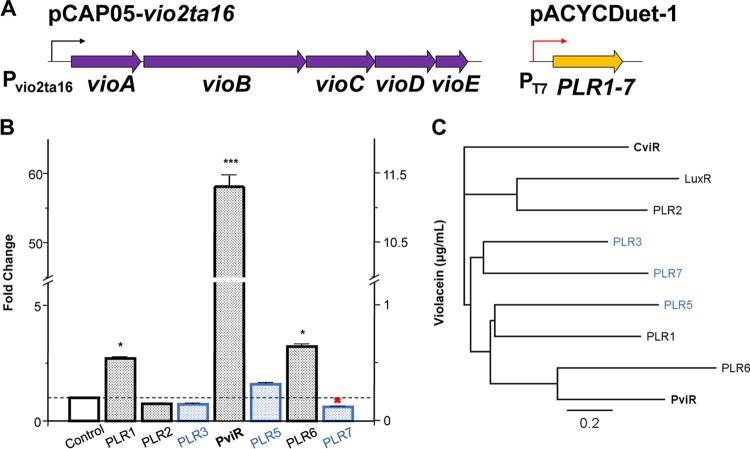
Coexpression of a LuxR-type receptor from *P. luteoviolacea* 2ta16 activates high levels of violacein production in *E. coli*. (A) Schematic illustrating coexpression of candidate quorum-sensing receptors from *P. luteoviolacea* with pCAP05-*vio2ta16* in *E. coli* BL21(DE3). (B) Quantification of violacein production in a coexpression experiment. Cultures were grown to an OD_600_ of 0.5 to 0.6, induced with 100 μM IPTG, and incubated at 18°C with shaking for 24 h before extraction and violacein quantification by HPLC. Candidate regulators are on the *x* axis and ordered by similarity to CviR as determined by DELTA-BLAST, and putative response regulators are blue. The data plotted are the mean ± standard error of total production on the right *y* axis and fold change compared to the control on the left *y* axis (*n* = 3). Statistical significance was determined with a one-tailed Student *t* test (*, *P* < 0.01; ***, *P* < 0.0005). PLR7 was not expressed solubly in *E. coli*, as indicated by the red X. (C) Neighbor-joining phylogenetic tree of quorum-sensing receptors LuxR and CviR and homologs identified from *P. luteoviolacea* 2ta16 generated with Geneious 5.1.7. An alignment was generated with a Blosum62 cost matrix, and the tree was built with a Jukes-Cantor genetic distance model in Geneious Tree Builder.

Previous studies have shown that quorum-sensing signal receptors are not soluble or active when expressed in the absence of stabilizing autoinducer ligands (30, 37). However, cloning and expression of the candidate regulators revealed that six out of seven could be expressed as soluble proteins in *E. coli* BL21(DE3) without the addition of any *P. luteoviolacea* extracts or purified AHL autoinducers (Fig. S6A). When the candidate regulators were coexpressed with pCAP05-*vio2ta16*, three receptors induced a statistically significant increase in violacein production in *E. coli* and one receptor, which we have named PviR, increased production nearly 60-fold compared to the control, resulting in production levels similar to those achieved by T7 promoter refactoring (11.12 ± 0.17 µg/ml, Fig. 4B). PviR does not cluster near *vio2ta16* or any identifiable autoinducer synthase genes, nor is it the most similar CviR homolog identified from *P. luteoviolacea* 2ta16 (Fig. 4C).

10.1128/mBio.01291-17.6 FIG S6  SDS-PAGE analysis of protein extracts of *E. coli* BL21(DE3) overexpressing candidate quorum-sensing signal receptors identified in native and heterologous hosts. Panels: A, *P. luteoviolacea* 2ta16; B, *E. coli*; C, *P. putida* KT2440; D, *A. tumefaciens* LBA4404; E, protein molecular size ladder used in all of the gels. Download FIG S6, PDF file, 0.3 MB. Copyright © 2017 Zhang et al. 2017 Zhang et al. This content is distributed under the terms of the Creative Commons Attribution 4.0 International license.

**FIG 5:**
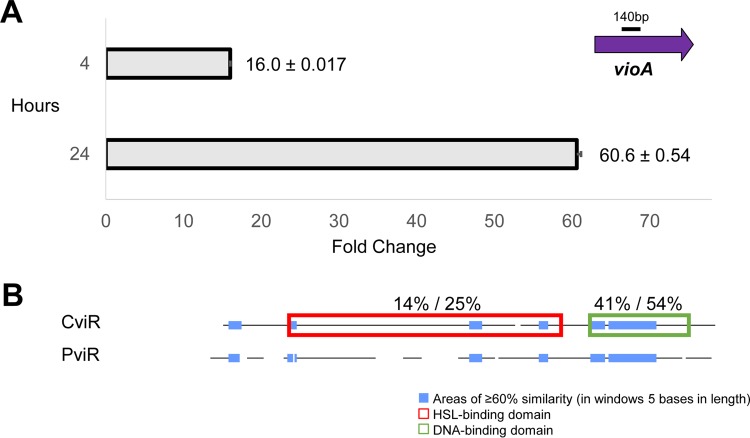
LuxR-type receptor PviR activates *vio2ta16* transcription independently of HSL autoinducers. (A) Fold changes in *vioA* transcript levels determined by qPCR. Changes were measured by the relative standard curve method with the *E. coli* gene *cysG* as the calibrator. Control and PviR coexpression cultures were grown as described previously (in the legend to Fig. 4) before total RNA isolation, DNase digestion, and cDNA generation with random hexamers for qPCR analysis. (B) Alignment of CviR and PviR sequences generated with Clone Manager. The values above the boxed domains are the amino acid identity/similarity percentages within those regions.

We verified that PviR controls violacein production at the transcriptional level by quantitative PCR (qPCR). Four hours after the induction of PviR in *E. coli* BL21(DE3), *vio2ta16* transcription increased 16-fold (Fig. 5A). After 24 h, we measured a 61-fold increase in *vioA* transcript levels, suggesting that the transcripts accumulated over time (Fig. 5A). This activity was not dependent on the addition of any exogenous culture extracts or compounds. PviR aligns poorly with CviR along the N terminus of the protein (where the AHL-binding domain is located) and is similar primarily within the C-terminal DNA-binding domain (Fig. 5B). Only one of six amino acid residues important for AHL binding is conserved (Fig. S5). These results indicate that PviR activation of *vio2ta16* transcription does not depend on an AHL autoinducer. Previously, it was reported that the LuxR-type transcription factor MalR from *Burkholderia thailandensis* activates a malleilactone promoter construct in *E. coli* independently of AHLs; however, MalR shows complete amino acid identity with AHL-binding residues (38). If PviR does require an autoinducer for transcriptional activation of the *vio2ta16* promoter, as is typical for quorum-sensing receptors, the molecule or a functional mimic is supplemented in LB medium or produced by *E. coli*. Recently, Bassler and coworkers showed that a ubiquitous autoinducer produced by *E. coli* can interact with a *Vibrio cholerae* quorum-sensing receptor to control the expression of genes involved in biofilm formation and toxin production (39).

**FIG 6:**
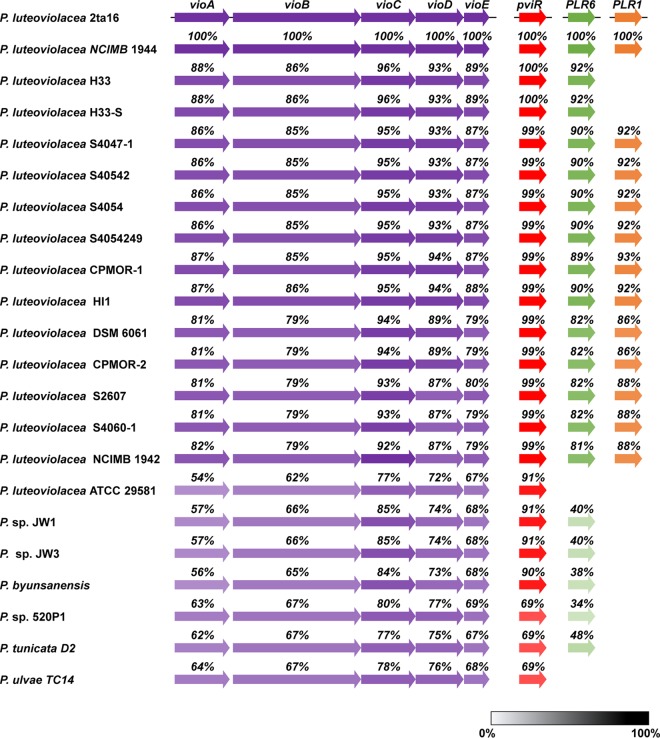
PviR is conserved across sequenced *Pseudoalteromonas* strains that possess the violacein BGC. Comparison of LuxR-type receptor conservation across all of the sequenced *Pseudoalteromonas* strains with the violacein BGC (*vioA* to *vioE*). All of the *Pseudoalteromonas vio* BGCs in the JGI and NCBI databases were identified by a protein BLAST search for VioC from *P. luteoviolacea* 2ta16 and referenced against BLAST hits for PviR, PLR6, and PLR1. Amino acid sequence identity percentages are listed above the arrows representing the genes and also indicated by color opacity.

Unlike PLR6 and PLR1, which also induced statistically significant, albeit smaller, increases in heterologous violacein production, PviR is conserved among all of the sequenced *Pseudoalteromonas* strains that possess the violacein BGC. As shown in Fig. 6, which lists 22 *Pseudoalteromonas* strains, including 16 strains of *P. luteoviolacea*, PLR6 is not found in 2 strains, while PLR1 is absent from 9. Interestingly, PviR is conserved at 91% amino acid identity in *P. luteoviolacea* ATCC 29581, even though the violacein biosynthetic genes have dropped to 54 to 77% identity. PviR appears to be specific to the genus *Pseudoalteromonas*. We identified the regulator in many *Pseudoalteromonas* species that lack the violacein gene cluster, excluding *Pseudoalteromonas atlantica*, which is phylogenetically distinct from other *Pseudoalteromonas* species (27). This pattern suggests that PviR has been conserved over evolutionary time while the violacein BGC diverged in sequence and was lost by many species. As PviR was characterized in *E. coli* and not in its native context, it may be part of a larger network that influences gene expression differently or more broadly across the entire *Pseudoalteromonas* genome, but this has not yet been explored.

### Host regulatory factors that influence heterologous violacein production.

**FIG 7:**
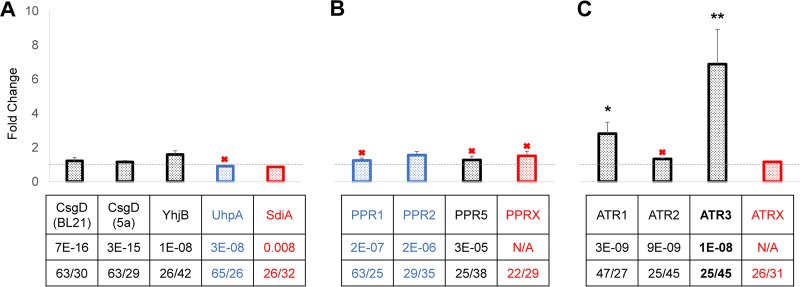
Host regulatory factors influence heterologous violacein production. Heterologous violacein production by *E. coli* BL21(DE3) through overexpression of PviR homologs from *E. coli* (A), *P. putida* KT2440 (B), and *A. tumefaciens* LBA4404 (C). Each table column lists the protein name, E value, and percent coverage/identity from top to bottom, respectively. Response regulators are blue. Regulatory proteins with E values below the significance threshold are red. Proteins that were not expressed solubly (see Fig. S6) are indicated by a red X. The data plotted are the mean ± standard error of the fold change in violacein production relative to the control in two independent experiments (total *n* = 5). Statistical significance was determined with a one-tailed Student *t* test (*, *P* < 0.05; **, *P* < 0.005).

Given that PviR upregulates violacein expression but does not cluster with *vio2ta16*, we speculated that host transcription factors must complement PviR-mediated promoter activation during heterologous expression. Thus, the availability or activity of compatible regulators could explain, in part, the observed differences in heterologous violacein production. Using PviR as a probe, we queried the genomes of the four host strains by DELTA-BLAST. Surprisingly, the most similar candidate identified was the regulatory protein CsgD from *E. coli*. Alignment of the sequences of PviR and CsgD revealed approximately 30% amino acid identity spanning 63% of the protein sequence, with an expect (E) value 6 to 9 orders of magnitude lower than that of the top candidates identified in *A. tumefaciens* and *P. putida* (Fig. 7A to C and S7). This result is perhaps reflective of the closer phylogenetic relationship between *P. luteoviolacea* and *E. coli* than the other two hosts.

A total of 11 PviR homologs were identified in *E. coli* (Fig. S7A). Although the genomes of *E. coli* DH5α and BL21(DE3) are very similar, seven of the BLAST hits are not identical proteins in the two strains, including CsgD, which contains two point mutations in BL21(DE3) compared to DH5α (S19P, A154V). A recent study revealed that metabolic physiology and gene expression can vary widely among different strains of *E. coli*, including BL21(DE3) and DH5α (40). Furthermore, the study showed that expression of *csgD* specifically is low in both strains under aerobic and anaerobic culture conditions. Thus, we hypothesized that overexpression of *csgD* or other PviR homologs in *E. coli* might rescue heterologous violacein production to levels that rival those in *P. putida* or *A. tumefaciens*.

10.1128/mBio.01291-17.7 FIG S7  Results of a DELTA-BLAST search querying the genomes of *E. coli* (A), *P. putida* KT2440 (B), and *A. tumefaciens* LBA4213 (C) with PviR from *P. luteoviolacea* 2ta16. The top panels show sequence alignments of homologs to PviR, displayed in order below the query sequence. The tables list BLAST hits, with genes selected for testing in bold and underlined, putative two-component system response regulators in blue, and hits with E values below the significance threshold in red. In panel A, K-12 homologs are shown in the top table and BL21(DE3) homologs are shown in the bottom table, with BLAST hits that differ highlighted in peach. Download FIG S7, PDF file, 0.6 MB. Copyright © 2017 Zhang et al. 2017 Zhang et al. This content is distributed under the terms of the Creative Commons Attribution 4.0 International license.

We selected the top three PviR homologs from *E. coli* and tested their ability to increase heterologous violacein production when overexpressed in *E. coli* BL21(DE3). We also selected a LuxR homolog that was not a PviR BLAST hit as a control. In total, five *E. coli* regulatory genes were tested, *csgD* from BL21(DE3) and DH5α; *yhjB* and *uhpA*, which are identical proteins in BL21(DE3) and DH5α; and the control, *sdiA*, which is present in DH5α but absent from BL21(DE3). Despite significant sequence similarity to PviR, neither CsgD variant was able to raise heterologous violacein production above the baseline. Although YhjB improved violacein production approximately 1.5-fold, this result was not statistically significant, as determined with a one-tailed Student *t* test (*P* < 0.05) (Fig. 7A). According to sodium dodecyl sulfate polyacrylamide gel electrophoresis (SDS-PAGE) analysis of proteins isolated at the time of extraction, UhpA was not overexpressed solubly under the conditions tested (Fig. S6B). Thus, we conclude that while *E. coli* regulatory proteins CsgD and YhjB are similar in sequence to PviR, they are not capable of activating the *vio2ta16* promoter. Although we cannot rule out the possibility that regulatory proteins not tested or identified are capable of activating *vio2ta16* expression, our results suggest that, despite their close phylogenetic relationship to *P. luteoviolacea*, laboratory *E. coli* strains are not able to express *vio2ta16* from the native pathway promoter.

As overexpression of the two CsgD variants from BL21(DE3) and DH5α produced very similar results, we reasoned that higher violacein titers from BL21(DE3) than from DH5α are the result of enhanced protein stability and not an increase in *vio2ta16* transcription. Differences in violacein expression could have arisen because of disparate activity or expression levels of transcription factors in the two strains; however, this does not seem to be the case. Furthermore, enhanced protein stability, which is a hallmark of BL21(DE3), is consistent with the finding that the violacein titers of BL21(DE3) were similar to those of DH5α at 30°C but higher at 18°C (Fig. 1).

Using the same methodology, we also identified and tested regulatory genes from *P. putida* KT2440 and *A. tumefaciens* LBA4404. Because violacein is readily expressed in these hosts, we hypothesized that we could identify PviR homologs capable of *vio2ta16* activation from these organisms. We identified 15 homologs from *P. putida* KT2440, 11 of which (including 4 of the top 5) are two-component system response regulators (Fig. S7B). The two most similar BLAST hits, PPR1 and PPR2, along with the most similar LuxR-type receptor, PPR5, were tested for the ability to increase violacein expression in *E. coli*. Additionally, a LuxR homolog, PPRX, which was not a BLAST hit for PviR, was selected as a control. However, only PPR2 was expressed solubly in *E. coli* BL21(DE3) (Fig. S6C) and did not induce a statistically significant increase in violacein production relative to the baseline (Fig. 7B). Addition of freeze-dried *P. putida* KT2440 culture supernatants to a concentration of 1 mg/ml did not alter the ability of any of the regulatory proteins tested to enhance violacein production (Fig. S9A). Thus, we were unable to conclusively identify transcriptional regulators from *P. putida* KT2440 that can activate *vio2ta16* expression.

10.1128/mBio.01291-17.8 FIG S8  Multiple-sequence alignment of CviR, LuxR, ATR1, and ATR3 generated with CLUSTAL O(1.2.4). Amino acid residues conserved across quorum-sensing signal receptors within the autoinducer- and DNA-binding domains are highlighted in yellow and gray, respectively. Download FIG S8, PDF file, 0.1 MB. Copyright © 2017 Zhang et al. 2017 Zhang et al. This content is distributed under the terms of the Creative Commons Attribution 4.0 International license.

However, testing of PviR homologs from *A. tumefaciens* LBA4404, also identified by DELTA-BLAST (Fig. S7C), revealed two regulators, ATR1 and ATR3, that increased violacein production approximately 3- and 7-fold, respectively (Fig. 7C), resulting in visibly purple cultures (data not shown). In particular, ATR3 raised violacein production to more than 2.5 µg/ml, which is far greater than the levels achieved by coexpression of the *P. luteoviolacea* LuxR-type receptor PLR1 or PLR6 (Fig. 4B). Although ATR3 is not able to raise *E. coli* violacein production levels to those of the *A. tumefaciens* heterologous host, we would not necessarily expect it to do so, as coexpression of PviR does not raise *E. coli* production to levels that rival those of *P. luteoviolacea*. Furthermore, ATR3 was only overexpressed at relatively low levels according to SDS-PAGE analysis (Fig. S6D). Confirmation of this LuxR-type receptor as a regulator of *vio2ta16* is consistent with the ability of the *A. tumefaciens* heterologous host to support robust production of violacein from the native pathway promoter. Unlike PviR, ATR3 does possess a conserved domain for AHL autoinducer binding, and four out of six AHL-interacting amino acid residues have been preserved (Fig. S8), although ATR3 activity and solubility were not dependent on the addition of exogenous AHLs. *A. tumefaciens* possesses a characterized quorum-sensing regulatory pair involving an AHL autoinducer and LuxR-type receptor TraR (36), but DELTA-BLAST searches with PviR as a probe did not detect TraR (Fig. S7C). Addition of freeze-dried *A. tumefaciens* LBA4404 culture supernatants to a concentration of 1 mg/ml did not alter the ability of any of the regulatory proteins tested to enhance violacein production in a statistically significant way (Fig. S9B). Because ATR3 is not the most similar PviR homolog identified, we conclude that it is difficult to predict the exact identity of compatible regulatory elements on the basis of sequence comparison alone. Furthermore, it has not escaped our attention that regulators that were not tested or identified from the hosts could also possibly influence heterologous *vio2ta16* expression.

10.1128/mBio.01291-17.9 FIG S9  Culture supernatants from *P. putida* and *A. tumefaciens* do not enhance receptor activity on the *vio2ta16* promoter in an *E. coli* expression system. Heterologous violacein production by *E. coli* BL21(DE3) through overexpression of PviR homologs from *P. putida* KT2440 (A) and *A. tumefaciens* LBA4404 (B) with addition of culture supernatants (PP and AT, respectively). Freeze-dried supernatants from log-phase *P. putida* KT2440 and *A. tumefaciens* LBA4404 cultures grown in LB at 30°C were dissolved in DMSO and added to *E. coli* BL21(DE3) coexpressing putative regulatory genes with *vio2ta16* at the time of IPTG induction. Freeze-dried LB medium prepared in the same way was also added as a control. The data are plotted as the mean ± standard error of the fold change compared to violacein production from *E. coli* BL21(DE3)/pCAP05-*vio2ta16*/pACYCDuet-1 treated with either control or culture supernatants. Significance was determined with a one-tailed Student *t* test (*n* = 2, *P* < 0.05). Addition of culture supernatants resulted in an increase in violacein production for only one regulatory gene, ATRX, which was not statistically significant (n.s.). Download FIG S9, PDF file, 0.1 MB. Copyright © 2017 Zhang et al. 2017 Zhang et al. This content is distributed under the terms of the Creative Commons Attribution 4.0 International license.

### Conclusion.

In this study, we validate the extension of our TAR cloning and heterologous expression platform to include Gram-negative proteobacterial expression hosts. Furthermore, our findings suggest that a more closely related host may not always be preferable for the heterologous expression of microbial BGCs. Heterologous antibiotic production remains challenging, particularly for pathways that utilize nonclustered and uncharacterized regulatory, biosynthetic, or resistance factors. For example, it was only recently discovered that glutamyl-tRNA^Glu^ is required for lantibiotic dehydration reactions during the biosynthesis of the antibiotic nisin (41); consequently, heterologous nisin production would require that host glutamyl-tRNAs be compatible with nisin biosynthetic machinery. Additionally, the antibiotic salinamide A inhibits bacterial RNA polymerase (RNAP) (42) and therefore must be expressed in hosts with resistant RNAP subunits. The phylogenetic relationship indicates a shared evolutionary history, and thus, it may theoretically provide a useful indication of host compatibility for the heterologous expression of BGCs in some cases. However, our study suggests that similarity is not directly synonymous with compatibility, either for an entire host organism or for an individual protein component. When working with challenging BGCs, broad-host-range heterologous expression represents a viable approach used to maximize the likelihood of achieving successful heterologous production.

Many characterized microbial BGCs are regulated by LuxR-type transcription factors, some of which are encoded by cluster-situated regulatory genes. Previous studies have shown that transcriptionally silent BGCs can be activated by the overexpression of genes encoding LuxR-type regulators (43, 44). Attempts to do so in a heterologous expression system would require the cognate autoinducer or binding ligand to be present and available in the host system. We identified a nonclustered LuxR-type regulator, PviR, that is capable of dramatic upregulation of *vio2ta16* expression independently of AHL autoinducers and is conserved among all of the sequenced *Pseudoalteromonas* host strains that harbor the *vio* BGC. Furthermore, we identified two PviR homologs from *A. tumefaciens* that are capable of robust activation of violacein production, consistent with the ability of the *A. tumefaciens* heterologous host to readily produce large amounts of violacein from the native pathway promoter. The cognate autoinducers, if they exist, for PviR and the *A. tumefaciens* homologs are unknown and may be produced by *E. coli*, which is not unprecedented (39).

Finally, although replacement of the *vio2ta16* promoter was an easy way to overcome host regulatory incompatibility with this pathway, most BGCs are not so easily refactored. The operonic architecture of BGCs can be complex and is usually only inferred on the basis of gene organization. Some BGCs may possess long operons or even single genes that cannot be reliably expressed from the T7 promoter (10, 45). Broad-host-range expression creates the possibility of achieving heterologous antibiotic production in the absence of pathway refactoring. Although only alphaproteobacterial and gammaproteobacterial hosts were tested in this study, earlier work on the RK2 replicon suggests that it is functional in a wide range of Gram-negative genera, although it is not clear whether bacterial phyla such as *Cyanobacteria*, *Spirochetes*, or *Bacteroidetes* have been systematically assessed (46). Furthermore, the maintenance of very large and repetitive multimodular BGCs presents a formidable challenge for heterologous expression in any system and must be evaluated in future work. We believe that the characterization and utilization of additional host organisms will aid the investigation of cryptic BGCs and could lead to unanticipated findings related to natural-product biosynthesis, regulation, or bioactivity.

## MATERIALS AND METHODS

### General methods and materials.

The primers and plasmids used in this study are reported in Table S1 in the supplemental material. Highly transformable *Saccharomyces cerevisiae* strain VL6-48N (*MAT*α *his3-D200 trp1-*Δ*1 ura3-*Δ*1 lys2 ade2-101 met14 psi*^*+*^* cirO*) was used as a host for TAR cloning. Yeast cells were grown in liquid YPD (yeast extract-peptone-dextrose) medium (2% d-glucose, 1% yeast extract, 2% peptone) supplemented with 100 mg/liter adenine and used for spheroplast preparation prior to TAR. Yeast transformants were selected for on synthetic histidine dropout (SD-His) agar containing 5-FOA (SD-Trp--5-FOA agar) consisting of 0.17% yeast nitrogen base without amino acids and ammonium sulfate (Sigma), 0.19% yeast synthetic dropout medium supplements without histidine (Sigma), 1 M sorbitol, 2% d-glucose, 0.5% ammonium sulfate, 100 mg/liter adenine, 2% agar, and 0.1% 5-FOA (Zymo Research). *E. coli* strains, *P. putida* KT2440, and *A. tumefaciens* LBA4404 were cultivated in LB medium (components purchased from BD Biosciences or Fisher Scientific) supplemented with the appropriate antibiotics. YM medium (yeast extract at 0.04%, mannitol at 1.0%, NaCl at 1.7 mM, MgSO_4 ⋅ _7H_2_O at 0.8 mM, K_2_HPO_4 ⋅ _3H_2_O at 2.2 mM) was used for transformation and preculture of *A. tumefaciens* LBA4404. *P. luteoviolacea* 2ta16 was grown in Marine Broth 2216 (BD Biosciences). For plates, 1.5% agar (Fisher Scientific) was added to the appropriate medium. DNA isolation and manipulations were carried out in accordance with standard protocols. DNA fragments larger than 3 kb were amplified with PrimeSTAR Max (Clontech Laboratories, Inc.); all other PCR products were amplified with PrimeSTAR HS DNA polymerase (Clontech Laboratories, Inc.).

10.1128/mBio.01291-17.10 TABLE S1  Primers and plasmids used in this study. Download TABLE S1, PDF file, 0.1 MB. Copyright © 2017 Zhang et al. 2017 Zhang et al. This content is distributed under the terms of the Creative Commons Attribution 4.0 International license.

### Construction and validation of pCAP05.

To capture all of the broad-host-range elements from pRK442(H) (20), fragments of 5,121 and 3,943 bp were amplified with primer pairs pCAP05-1_5121F/R and pCAP05-2_3943F/R (Table S1; Fig. S1A), respectively. In parallel, a 2,024-bp fragment containing *CEN6_ARS4* and *HIS3* and a 1,639-bp fragment containing *pADH* and *URA3* were amplified from pARS-VN (47) with primer pairs pCAP05-3_2024F/R and pCAP05-4_1639F/R (Table S1; Fig. S1A), respectively. The four PCR-amplified fragments were combined and assembled into a single construct with Gibson Assembly (New England Biolabs) to generate pCAP05. The circular construct was digested with NdeI for verification and transferred to *S. cerevisiae* VL6-48N and selected against or for on histidine-deficient agar plates with or without 5-FOA (Fig. S1B to D). pCAP05 was transferred to *P. putida* KT2440 and *A. tumefaciens* LBA4404 by electroporation at 2,500 V with a 2-mm cuvette. To prepare electrocompetent *P. putida*, cells grown at 30°C to an optical density at 600 nm (OD_600_) of 1.0 were washed twice with an ice-cold 10% glycerol solution and resuspended in a small volume of the glycerol solution before the addition of up to 1 μg of DNA. Separately, electrocompetent *A. tumefaciens* was prepared by washing cells grown at 30°C in YM medium to an OD_600_ of 0.5 with ice-cold 1 mM HEPES buffer (pH 7.5) twice before resuspension in 1 mM HEPES--10% glycerol and the addition of 100 μg of DNA. *P. putida*/pCAP05 was recovered for 2 h at 30°C in LB medium before selection on LB agar supplemented with 15 μg/ml tetracycline (Fig. S1E). *A. tumefaciens*/pCAP05 was recovered for 3 h in YM medium before selection on YM agar supplemented with 5 μg/ml tetracycline (Fig. S1F).

### TAR cloning of *vio2ta16*.

The 9-kb DNA region containing *vio2ta16* was PCR amplified from the genomic DNA of *P. luteoviolacea* 2ta16 in three fragments (each approximately 3 kb) with primer pairs vio2ta16-1_3121-40F/vio2ta16-1_3121-134R, vio2ta16-2_3058-134F/vio2ta16-2_3058-108R, and vio2ta16-3b_3027-482F/vio2ta16-3b_3027-40R (Table S1; Fig. S2A). These fragments were cloned into pCAP05 in *S. cerevisiae* VL6-48N by a previously reported protocol (15), with minor modifications. *S. cerevisiae* VL6-48N was grown to an OD_600_ of 0.7 to 1.0 in 50 ml of YPD medium supplemented with adenine (100 mg/liter) at 30°C with shaking. The cells were harvested and washed with ice-cold water and osmotically stabilized in 1 M sorbitol at 4°C overnight prior to spheroplast preparation. Preparation of spheroplast cells was carried out with a lytic enzyme (Zymolyase 20T; MP Biomedicals) at a final concentration of 0.1 mg/ml with 30 to 40 min of incubation. A 0.5-μg sample of each PCR product and ClaI/XhoI-linearized pCAP05 were added to spheroplast cells, and the transformation was mediated by PEG 8000 (Sigma). The transformed spheroplasts were mixed with 10 ml of SD-His top agar (containing 3% agar) at 55°C and overlaid on SD-His--5-FOA agar. The plates were incubated at 30°C for 3 days. Hundreds of colonies appeared per plate, and four were picked, transferred onto new SD-His agar plates, and incubated for 24 h at 30°C. Cells were lysed with Zymolyase 20T at 37°C for 30 min and subsequently boiled at 98°C for 5 min. The captured *vio2ta16* BGC was screened with primer pair vio2ta16check_402F/R (Table S1). Plasmids were extracted from PCR-positive clones and transferred into *E. coli* Top 10 cells by electroporation. The plasmids were purified from tetracycline-resistant *E. coli* clones, and the resulting constructs were confirmed by restriction analysis; the vector containing *vio2ta16* BGC was designated pCAP05-*vio2ta16* (Fig. S2B).

### Heterologous expression and quantification of violacein production.

pCAP05-*vio2ta16* was introduced into *E. coli*, *P. putida*, and *A. tumefaciens* by electroporation as described above. Precultures were grown overnight at 30°C in LB (*E. coli* and *P. putida*) or YM (*A. tumefaciens*) medium supplemented with 5 μg/ml tetracycline. YM was used for *A. tumefaciens* precultures to prevent cell clumping. All of the cultures were seeded the next day with a standard inoculum of each strain to give a starting OD_600_ of 0.02 in LB with 5 μg/ml tetracycline. One-milliliter cultures were then incubated for 48 h with shaking at 220 rpm at 18 or 30°C before cells were harvested by centrifugation and extracted with methanol. For HPLC analysis, samples were injected onto a Phenomenex Kinetex XB-C18 reversed-phase HPLC column (2.6 μm, 150 by 4.6 mm [inside diameter]) and analyzed with an Agilent 1260 liquid chromatography system by gradient elution (A, CH_3_CN with 0.1% trifluoroacetic acid [TFA]; B, H_2_O with 0.1% TFA; 50 to 100% A over 5 min and 100% A from 5 to 8 min at 0.7 ml/min) at 575 nm. Violacein was quantified with a standard purchased from AdipoGen Life Sciences (San Diego, CA) with the formula *y* = 3238.1*x* − 33.266, where *x* is the concentration and *y* is the integrated UV absorbance at 575 nm (*R*^2^ = 0.9988). Data were collected from two independent experiments, each time from triplicate cultures.

Wild-type *P. luteoviolacea* strain 2ta16 was grown in MB medium without any antibiotics. Violacein production was quantified as described above, and data were collected from three independent experiments, each time in triplicate.

### *vio2ta16* promoter replacement and testing.

pET28a was amplified with primer pair pET28a-vioE-F/pET28a-vioA-R (Table S1), and the product was purified and digested with DpnI. The pCAP05 backbone was replaced in *E. coli* BW25113/pIJ790/pCAP05-*vio2ta16* with the PCR targeting system (29). The resulting plasmid, pET28a-*vio2ta16*, was confirmed by restriction analysis (Fig. S2C) and sequencing (Genewiz, San Diego, CA) and transformed into *E. coli* BL21(DE3). A fresh colony was picked and inoculated into LB with 50 μg/ml kanamycin and grown overnight at 37°C. Precultures were reinoculated into fresh LB with the same antibiotic and grown at 37°C to an OD_600_ of 0.5 to 0.6. Isopropyl-β-d-thiogalactopyranoside (IPTG) was added to a 0, 10, 100, or 1,000 μM final concentration before incubation at 18 or 30°C with shaking (220 rpm) for 24 h. Violacein production in 1-ml cultures was quantified by HPLC as described above. Two independent experiments were conducted, each time with triplicate cultures grown under each condition.

### Cloning and testing of regulatory genes from *P. luteoviolacea* 2ta16.

PLR1 to PLR7 were amplified with the primer pairs listed in Table S1. PCR products and pACYCDuet-1 were digested with the restriction enzyme pair NcoI/HindIII or NdeI/XhoI (as indicated in Table S1) and ligated with T4 DNA ligase (New England Biolabs). The resulting constructs were screened by restriction digestion, verified by sequencing, and transferred to BL21(DE3)/pCAP05*vio2ta16*. BL21(DE3)/pCAP05-*vio2ta16* with the empty vector was used as a control. Single clones were picked for precultures grown overnight at 37°C in LB medium supplemented with 15 μg/ml tetracycline and 34 μg/ml chloramphenicol. One hundred microliters of each preculture was transferred to 10 ml of fresh LB with the same antibiotics and grown at 37°C to an OD_600_ of 0.5 to 0.6. Cultures were induced with 100 μM IPTG and incubated for an additional 24 h at 18°C with shaking (220 rpm). Violacein production was again quantified by HPLC as described above. Protein expression was checked by SDS-PAGE.

### Cloning and testing of regulatory genes from *E. coli*, *P. putida*, and *A. tumefaciens*.

PviR homologs from *E. coli*, *P. putida*, and *A. tumefaciens* were cloned and tested as described above. Violacein production was again quantified by HPLC, and protein expression was checked by SDS-PAGE in two independent experiments. To determine whether small molecules secreted by *P. putida* and *A. tumefaciens* influence the ability of tested regulatory genes from the two hosts to enhance violacein production, 50 ml of culture supernatants from *P. putida* and *A. tumefaciens* grown to an OD_600_ of approximately 1.0 in LB were freeze-dried, resuspended in dimethyl sulfoxide (DMSO), and added to *E. coli* BL21(DE3) expression cultures to a final concentration of 1 mg/ml at the time of IPTG induction. Freeze-dried LB was added as a control, and violacein production by all of the cultures was quantified by HPLC.

### qPCR analysis.

Single clones of BL21(DE3)/pCAP05-*vio2ta16* with pACYCDuet-1-*pviR* and empty pACYCDuet-1 were picked and grown as described above for testing of violacein induction activity. Cells were harvested 4 and 24 h after IPTG induction for RNA isolation. Cells were stabilized with RNAprotect Cell Reagent (Qiagen). RNA was isolated with an RNeasy minikit (Qiagen), and cell lysis was obtained by homogenization with the FastPrep system (BIO 101 Inc.) (five cycles of 30 s at 5.5 speed). Turbo DNase (Thermo Fisher Scientific) treatment was carried out for 2 h in accordance with the manufacturer’s instructions. The RNA was checked on an agarose gel, and the absence of genomic DNA in the sample was confirmed by PCR with primers for the housekeeping gene *cysG* (48). cDNA synthesis was carried out with SuperScript IV reverse transcriptase (Thermo Fisher Scientific), 50-pg/μl random hexamers, and 500 ng of RNA in accordance with the manufacturer’s protocol. The relative standard curve method was used for relative quantification of *vioA* transcripts during PviR coexpression compared to the control. BL21(DE3)/pCAP05*vio2ta16*/pACYCDuet-1-*pviR* genomic DNA was used to generate 8-fold dilution series standard curves with the primers for *vioA* (qRT-vioA_140F/R) and the calibrator *cysG* (qRT-cysG_126F/R) listed in Table S1. The standard curves were run in triplicate, and each cDNA reaction was run in parallel five times, along with reverse transcriptase-negative cDNA and no-template control reactions. The qPCR mixtures contained each primer pair at 0.5 μM, 1 μl of 10-fold-diluted cDNA, and 1× SYBR green PCR master mix (Thermo Fisher Scientific) for a total volume of 10 μl. A Stratagene Mx3000p qPCR thermocycler was used with a cycling protocol consisting of 1 cycle of 50°C for 2 min; 1 cycle of 95°C for 2 min; 40 cycles of 95°C for 15 s and 60°C for 1 min; and 1 cycle of at 95°C for 1 min, 55°C for 30 s, and 95°C for 30 s to generate a melting curve.
