# Supplementary figures and images for: Broad-Host-Range Expression Reveals Native and Host Regulatory Elements That Influence Heterologous Antibiotic Production in Gram-Negative Bacteria
Source: mBio. 2017 Sep 5;8(5):e01291-17. doi: 10.1128/mBio.01291-17 (PMC5587914; doi:10.1128/mBio.01291-17)

**A**

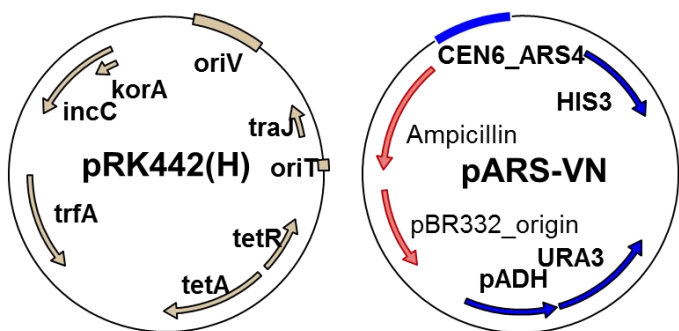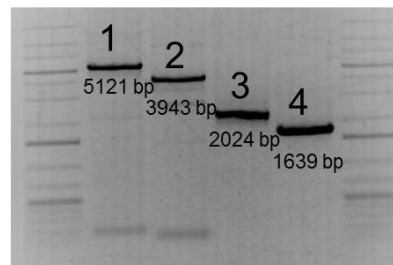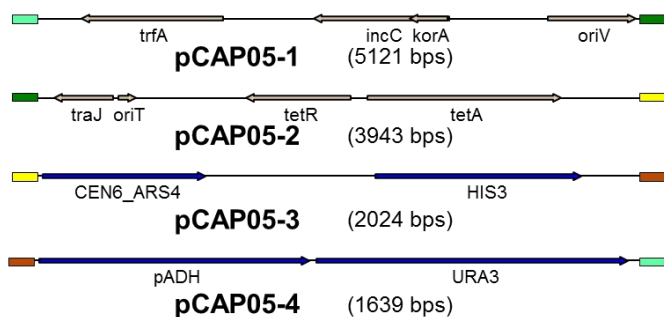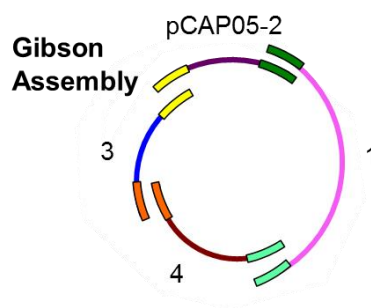

**B**

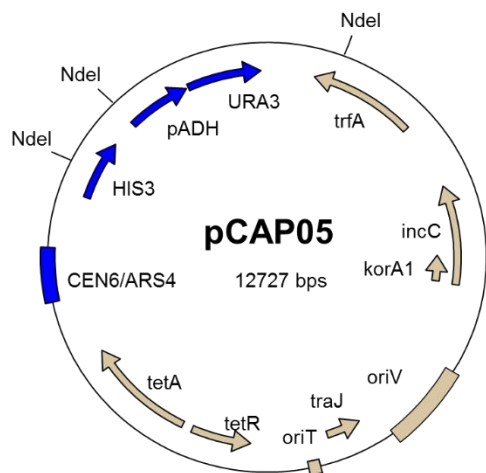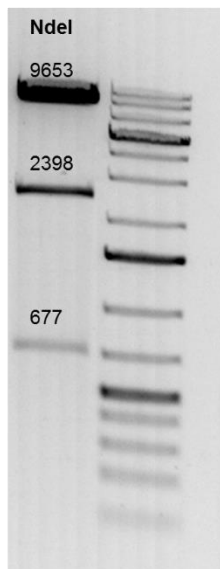

**C**

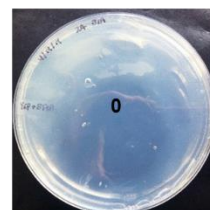

**D**

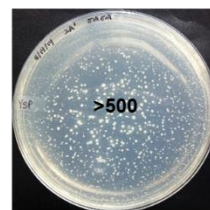

**E**

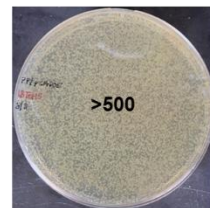

**F**

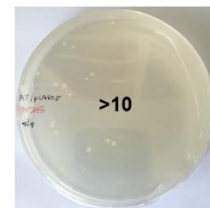

Supplement: FIG S1 [file mbo004173462sf1.pdf]

**A**

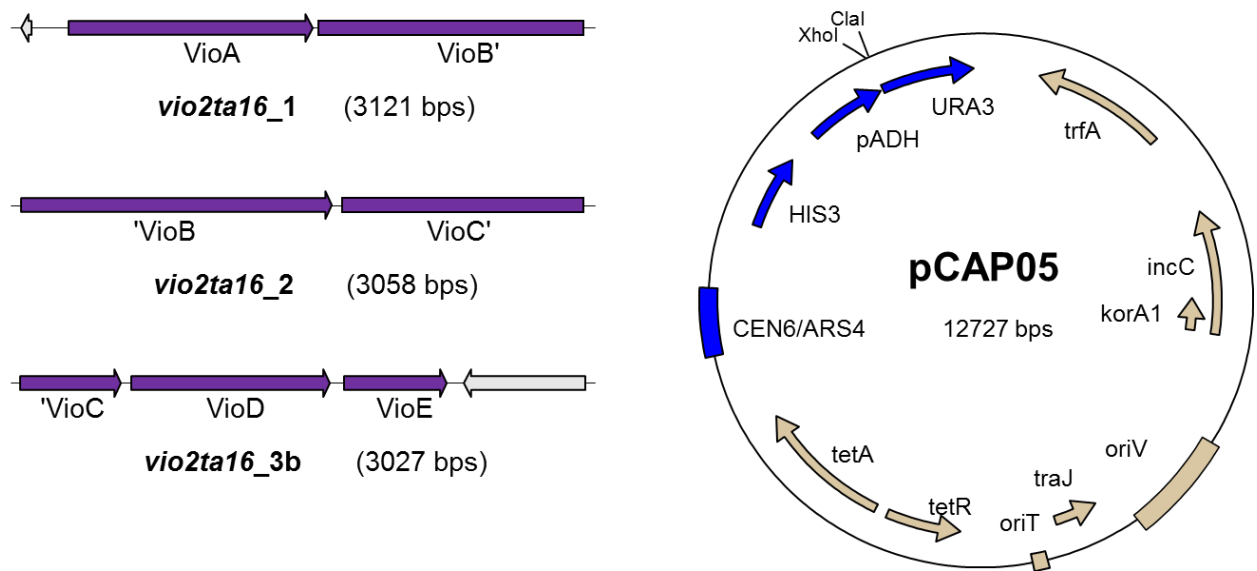

**B**

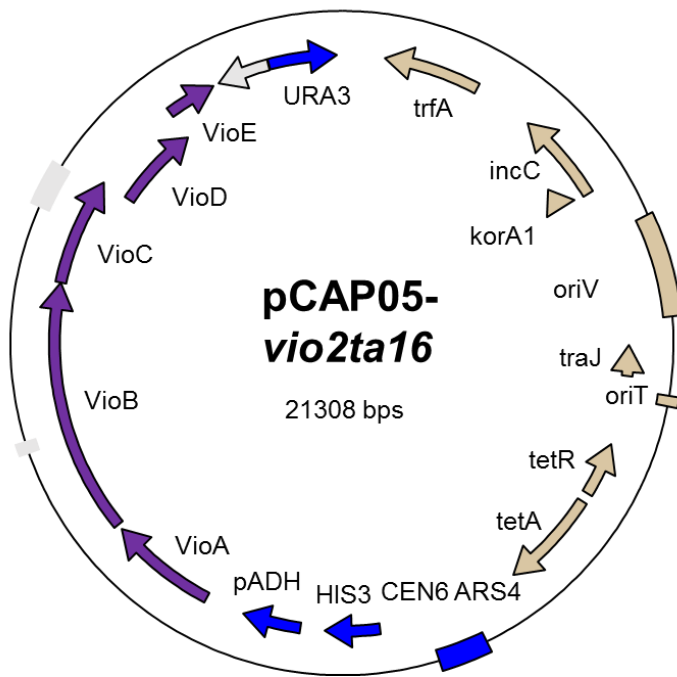

| NdeI |
|------|
| 9653 |
| 6211 |
| 3280 |
| 1487 |
| 677  |

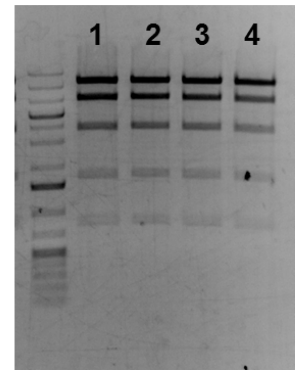

C

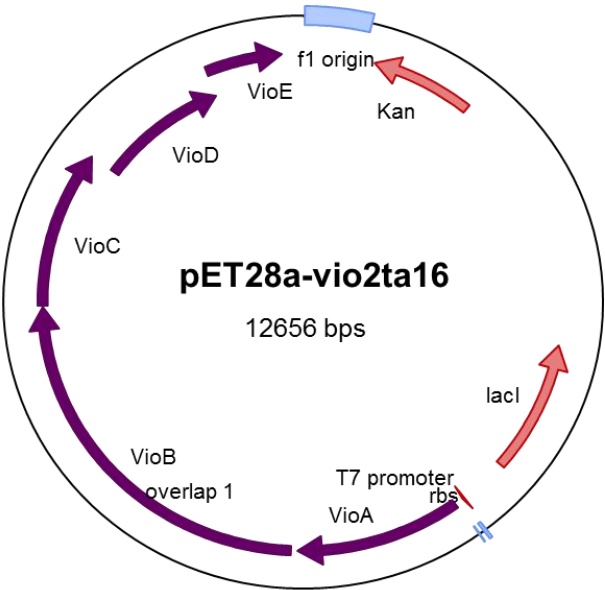

| Dral | EcoRV | NcoI |
|------|-------|------|
| 9869 | 7489  | 7205 |
| 1130 | 3760  | 5451 |
| 1042 | 1143  |      |
| 576  | 239   |      |
| 39   | 25    |      |

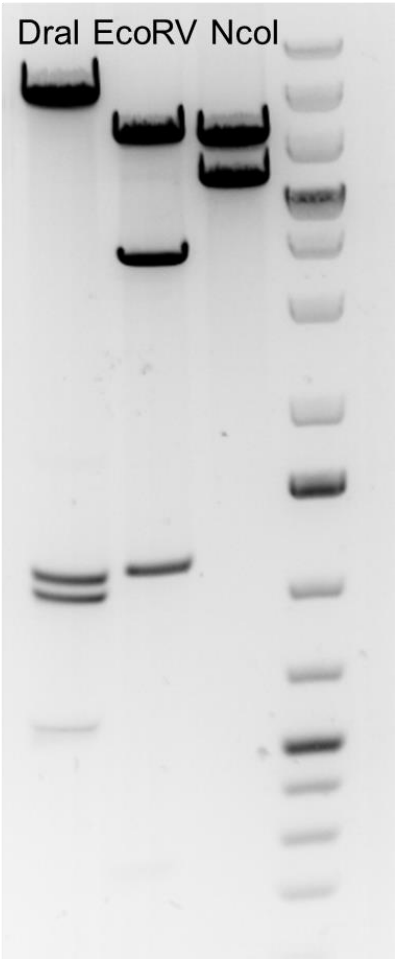

Supplement: FIG S2 [file mbo004173462sf2.pdf]

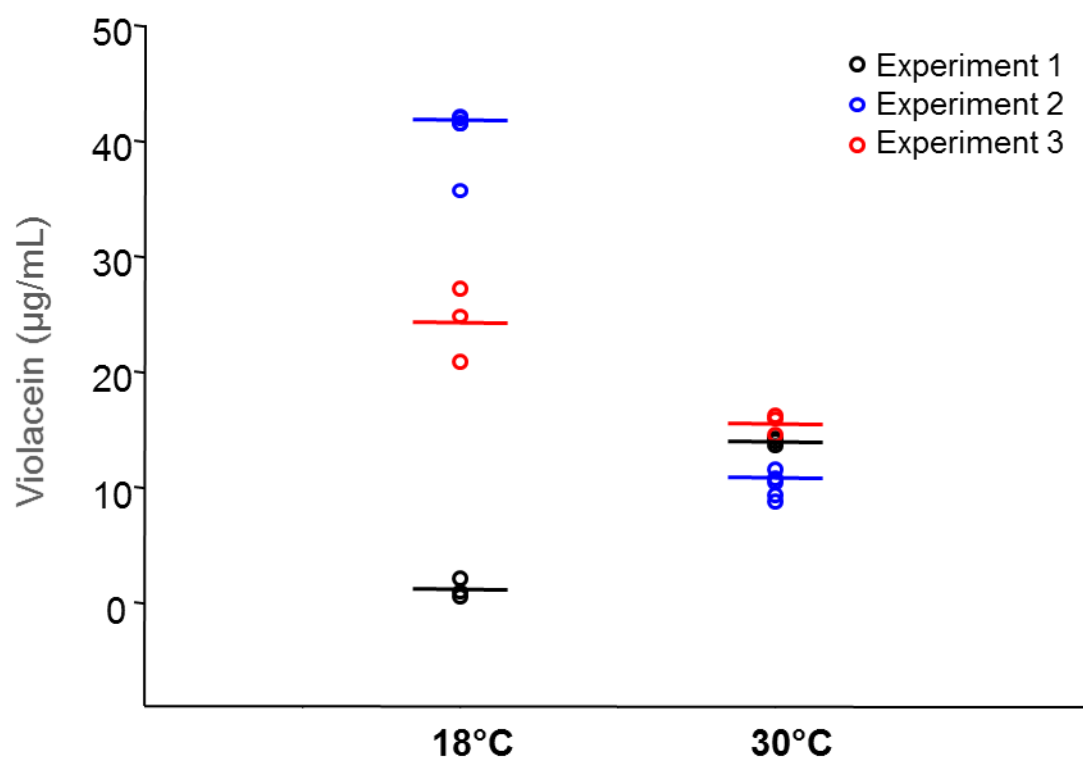

Supplement: FIG S3 [file mbo004173462sf3.pdf]

**A**

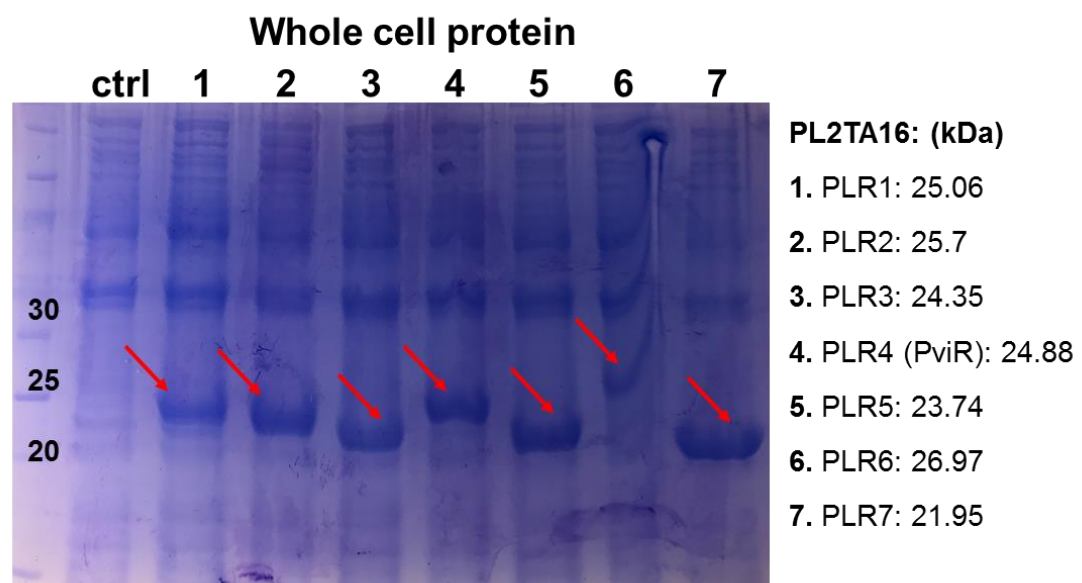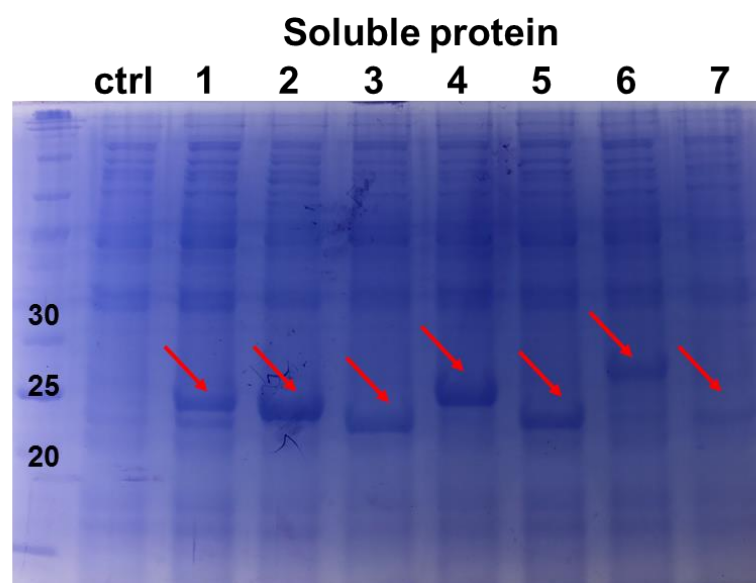

**B**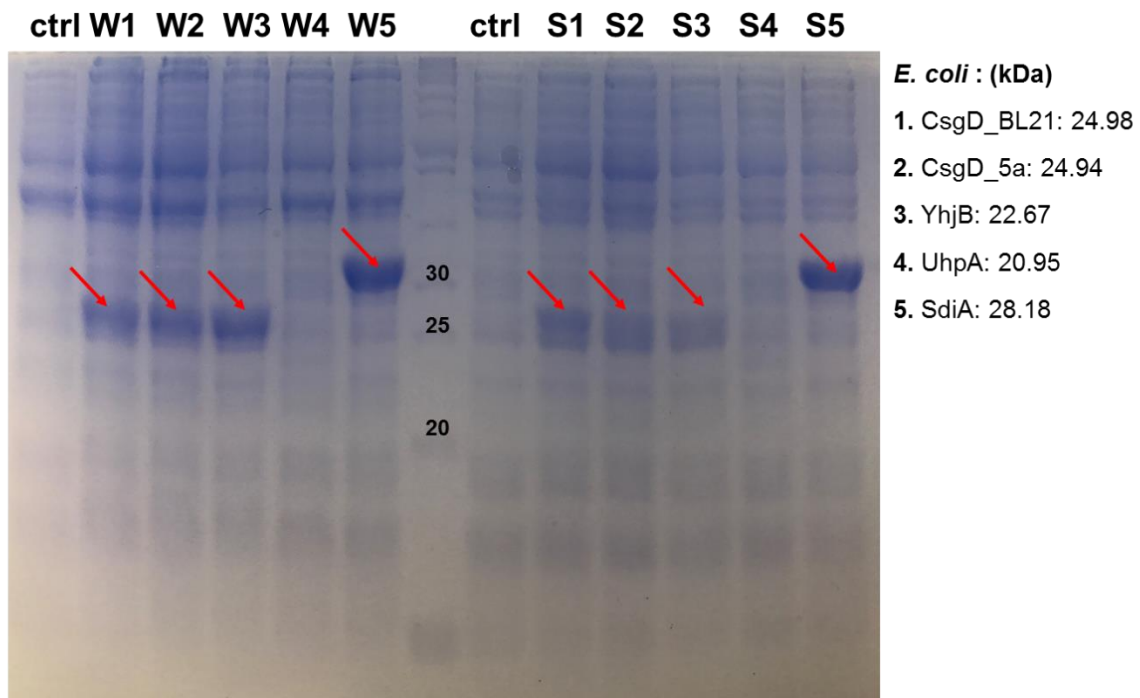**C**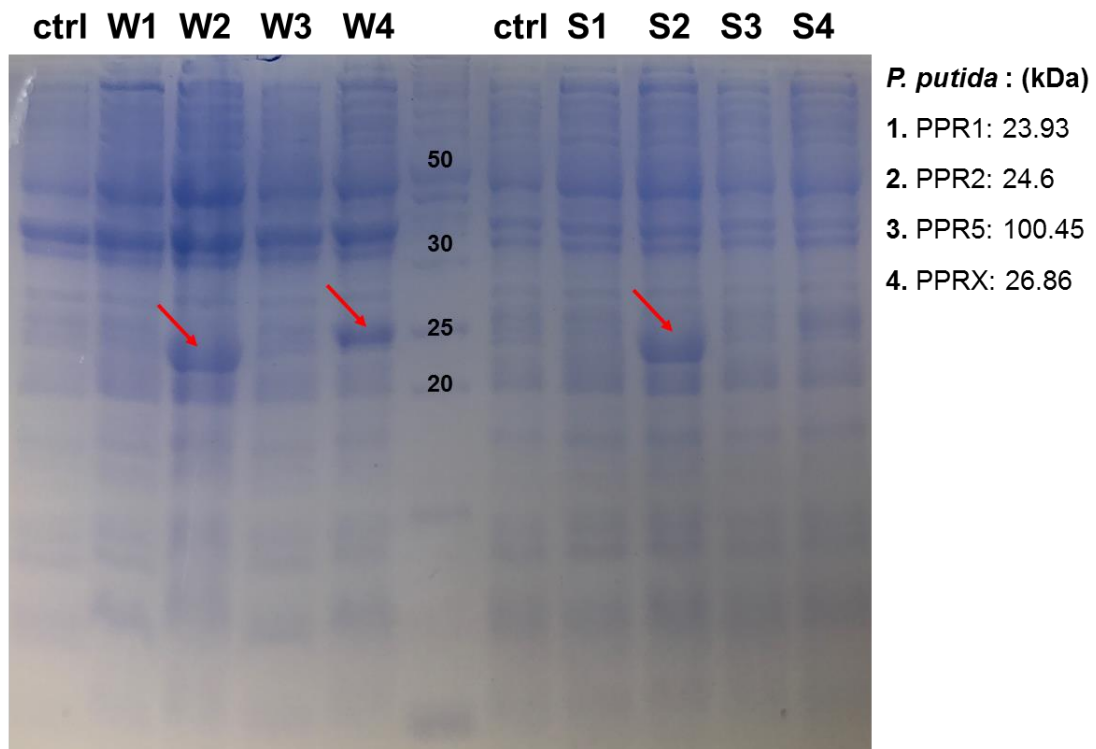

**D**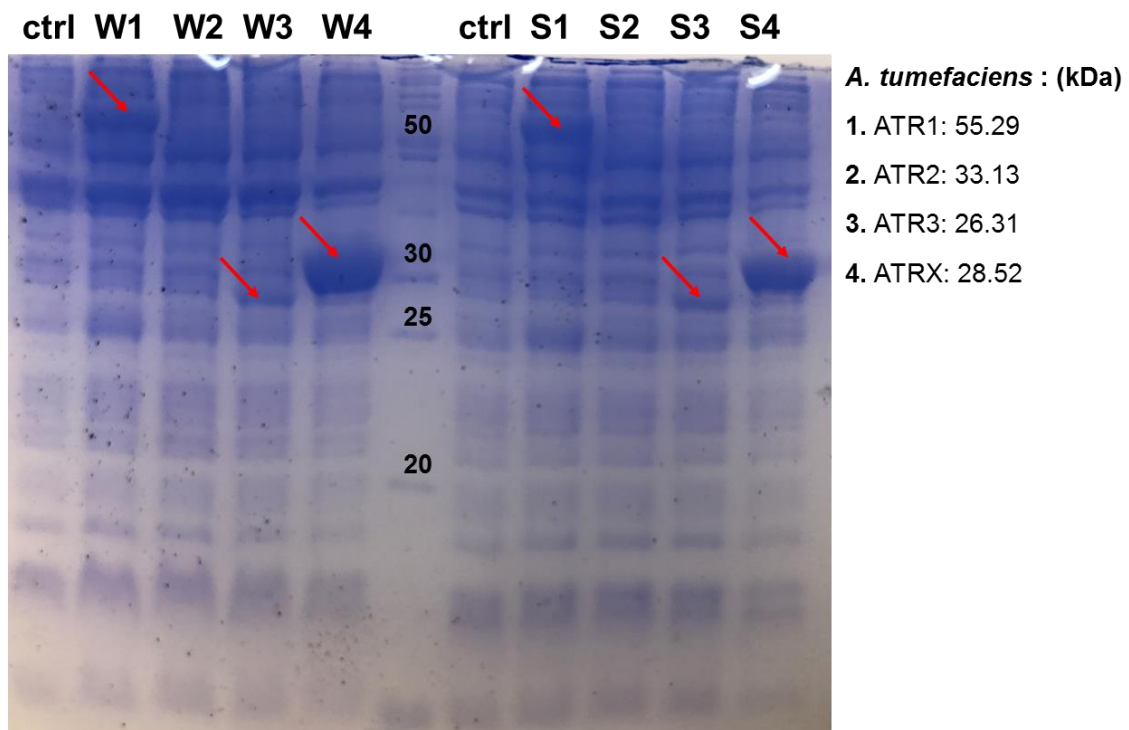**E**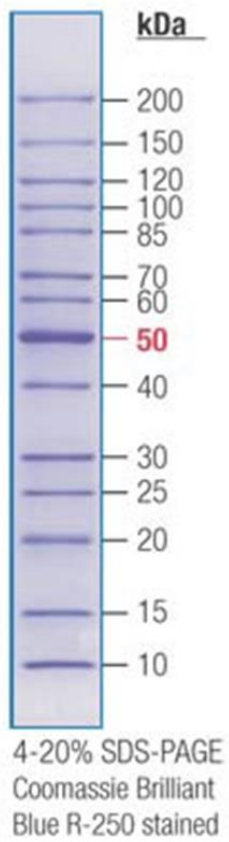

Supplement: FIG S6 [file mbo004173462sf6.pdf]

**A**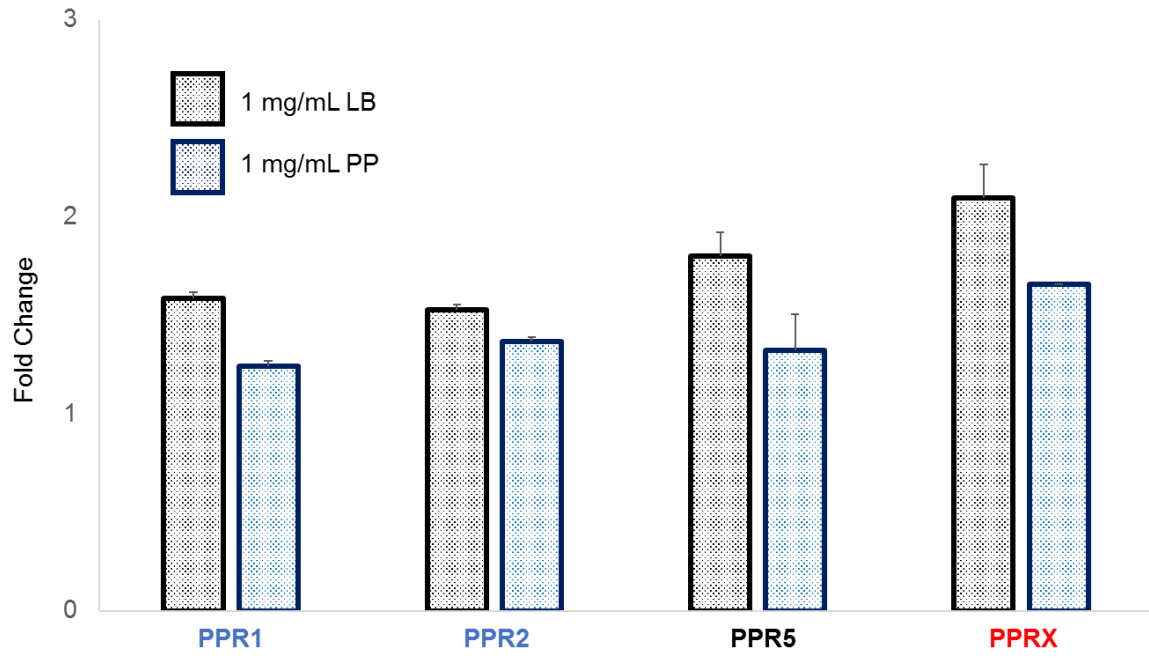**B**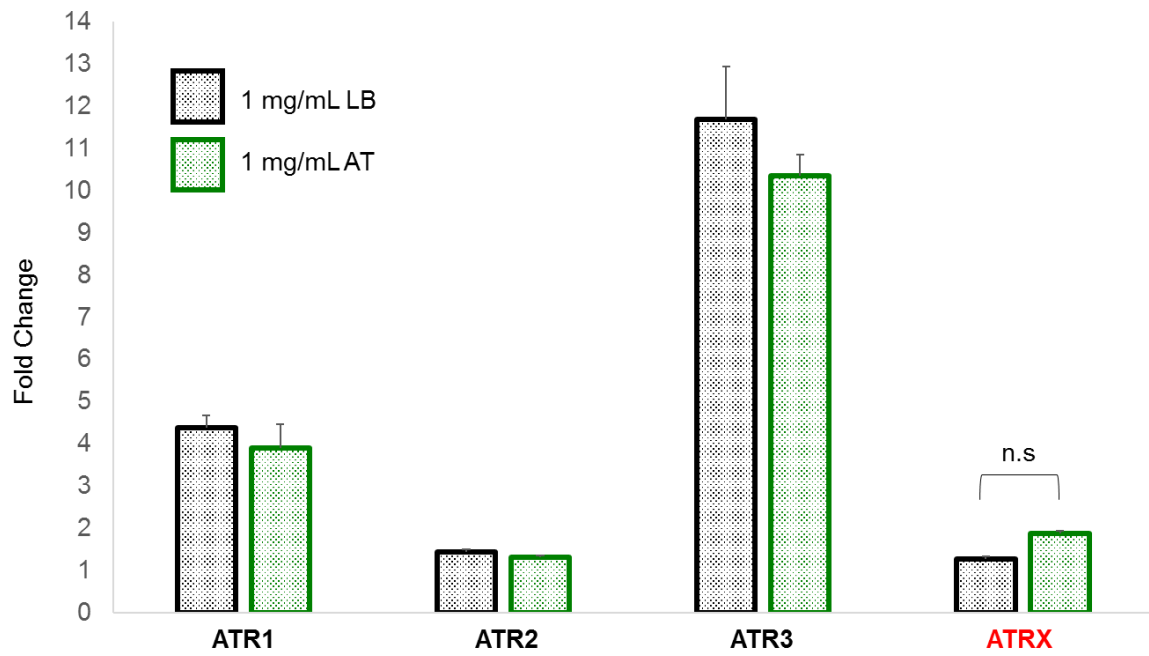

Supplement: FIG S9 [file mbo004173462sf9.pdf]
