# Supplementary material for: Broad-Host-Range Expression Reveals Native and Host Regulatory Elements That Influence Heterologous Antibiotic Production in Gram-Negative Bacteria
Source: mBio. 2017 Sep 5;8(5):e01291-17. doi: 10.1128/mBio.01291-17 (PMC5587914; doi:10.1128/mBio.01291-17)
Supplement: FIG S4 [file mbo004173462sf4.pdf]

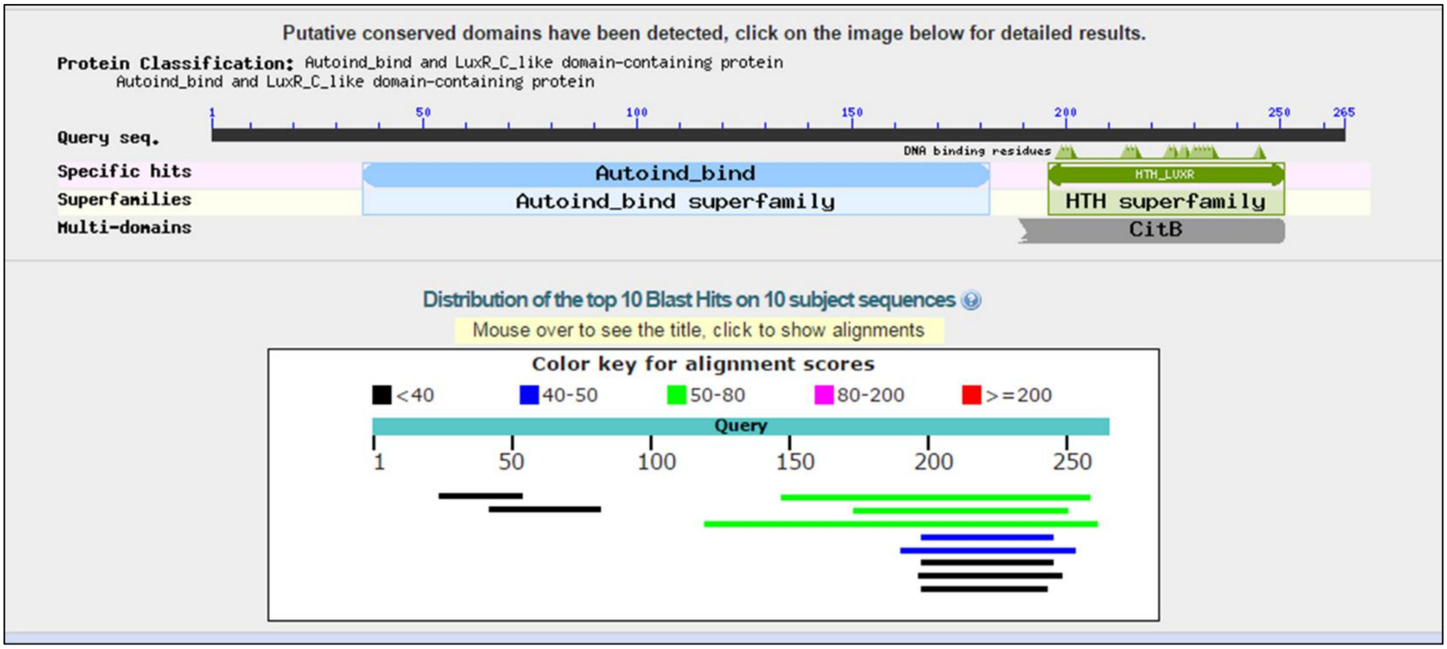

Name	Locus Tag (PL2TA16_)	Score	Coverage (%)	E value	Identity (%)
PLR1	01227	73.9	41%	2e-16	36%
PLR2	00118	66.2	29%	9e-14	44%
PLR3	03988	56.2	53%	3e-10	30%
PLR4 (PviR)	04751	43.1	18%	9e-06	48%
PLR5	01039	40.4	23%	7e-05	33%
PLR6	04047	38.5	18%	3e-04	40%
PLR7	00814	38.1	19%	4e-04	40%
