# Supplementary material for: Broad-Host-Range Expression Reveals Native and Host Regulatory Elements That Influence Heterologous Antibiotic Production in Gram-Negative Bacteria
Source: mBio. 2017 Sep 5;8(5):e01291-17. doi: 10.1128/mBio.01291-17 (PMC5587914; doi:10.1128/mBio.01291-17)
Supplement: FIG S5 [file mbo004173462sf5.pdf]

```
PviR    ------------------------------MGSS-----------TAFLL----------    9
PLR6    ------------------------------MGKAIQTSN----KEVSLFV----------    16
PLR5    ------------------------------MGNGKENSL--------TIL----------    12
PLR3    ------------------------------------MGN--------QFL----------    6
PLR7    --------------------------------------M--------KIL----------    4
LuxR    ----------------------------------------MNIKNINANEKIIDKIKTCN    20
PLR2    ------------------------------MGKPTEQDSYNPFKDGESIRRIIDLFDSIH    30
CviR    MVTSKPINARPLPAGLTASQQWTLLEWIHMAGHIETEGE------LKAFLDN--------    46
PLR1    ------------------------------MGFLGCTKE------LSSVLKAIELLKTSI    24

PviR    -------------LDKEPINTIGINV--LQPLLKTQGLDVVTGTDISEVPE--D------    46
PLR6    -------------LTHNKTDTQDFEFIRFVKILEATSNQIKIDTRLPDTPLRAQ------    57
PLR5    IADD--HHLVRQGL---------------RSLIERSQTQYNI-SDVATGEQAWQFISQY-    53
PLR3    IADD--HPLFREAL---------------KGALQNQFEGLEVIES-ENFEQTLERLSQYD    48
PLR7    VVED--QALVRNAI---------------SALLSLE-ANLEVVGQAEDGQQALEY-LANH    45
LuxR    NNKDINQC-LSEIAKIIHCE--YYLFAIIYP-HSIIKPDVSI---IDNYPEKWRK--YYD    71
PLR2    HADDLKEA-LTTIARDVGYD--CLAFVDYSP-LPNHIQPVQV---YGHYQEELSV--LFE    81
CviR    ------------ILSQAPSDRIILVLGRLNNQNQIQRMEKVL---NVSYPSDWLN--QY-    88
PLR1    IERSLDGIVFQEAMSFAESDELYLALIDKS---SL-EVKTEC---LRSFPQDFKE--DLL    75

PviR    -----TRLLFIETAVNDA-WGKLKEQ-LVNLKVSCDIVLFNLDENPELANRALLSGIR-G    98
PLR6    -----HHLYLVDISHREC-QDLLSAE-VSALAAQQNVLLFNAQPSLVNEQTALLARIK-G    109
PLR5    Q----PDLAILDIAMGDL-SGLKVCEHVKQRKLKTRIIFLSMHDDIKVIHRAFEVGAD-A    107
PLR3    D----LDLLLLDLHMPGN-GDLYGLIRIREDHPSLPIVVVSGSEDLNVISKVMGYGAM-G    102
PLR7    E----PDIVLSDIEMPNV-TGLELAQIIQEKYPRVKVVIMTTFSRAGYIRRAMAADVK-G    99
LuxR    DA----GLLEYDPVVDYSKSHHSPIN-WN-----VFEKKTIKKESPNVIKEAQESGLITG    121
PLR2    S----------DKVLAHSKSGIRLCS-LA-----KLTGALNIAESLHV------------    113
CviR    ---SQENFAQHDPIMRIH-LGQGPVI-WE-----ERFSRAKGSEEKRFIAEASSNGMGSG    138
PLR1    TVAKVKKFTNINILKSLC-KDR--------------------------------NPLSDI    102
                   :

PviR    VFYTTDNAD-------------VLMKGIRLLMEDQLWYRREIMCNALNRMLQFNKDA--L    143
PLR6    VIYQNTSAE-------------NIFKGIQRVLNGELWFCRTSISQAFNELIQQIPNIPRP    156
PLR5    Y-------------LSKSEAFDTLNQALQTVAAGHSFI-SPSIESELARYKAAS------    147
PLR3    F-------------IPKASSSQDIVSALQQVLDGENWL-PADIKEKINDLDGED------    142
PLR7    F-------------ILKEAPSDYLVNALKKISVGQKVI-DPELAM--NALDDS-------    136
LuxR    FSFPIHTASNGFGMLSFAHSDKDIYTDS--LFLHASTNVPLMLPSLVDNYQKIN------    173
PLR2    --LPLRGIKGIIGALVFNVPC-DLAHKV--TVEQVDWYWTILSPALLN----AA------    158
CviR    ITFSAASDRNNVGSILSIGGKEPGRNAA--LVAMLNCLTPHLHQAAVRIA-NL-------    188
PLR1    TIYDLDNPNSNFLTLVAFNNKRSNRTAP--ASYLIELVLPYLHKAQISRYQND-------    153

PviR    HKLTEGDIEPVKLTKREKAIISLMSKGAKNKEIAEDLNISPHTVKTHLYSAFRKTKCRNR    203
PLR6    QSLDIQDSELELLTAREKSVIKLLASGAKNDDIADSLNISSHTVKTHIYSAFKKTNSRNR    216
PLR5    ---T-----QFLLTAREKQIVSYITQGKSNRQIADTLCISIKTVDNHRTKAMRKLGVNKA    199
PLR3    ---RELAQQIASLTPQQYKVLQYLHEGLLNKQIAYELNISEATVKAHITAIFRKLGVYNR    199
PLR7    ----------DPLSDKERKALRLASEGMKTRQIAESLFLSEGTVRNYLSDAIAKLNATNR    186
LuxR    ---TTRKKSDSILTKREKECLAWASEGKSTWDISKILGCSERTVTFHLTNTQMKLNTTNR    230
PLR2    ---LRCRKDHFNITKRERDCVLWASEGKTSWEISQILGITERTVNFHLTNCIEKTQSANR    215
CviR    ---PPASPSNMPLSQREYDIFHWMSRGKTNWEIATILNISERTVKFHVANVIRKLNANNR    245
PLR1    ---KTSRSPIQSLTNREKEVLDWISSGKTNGEIGMILGISQYTVKNHVAKILEKLNAPNR    210
                    :: ::   .     *  . :*.  *  :  **  :      *    :

PviR    IELLSWAQQNIPDEIR*-------    219
PLR6    IELANWAQKHIPLNSAPVSIQRH*    239
PLR5    AELVKYGLEEGLVV*---------    213
PLR3    TQAVLIASKLQLEPIEAAK---*-    218
PLR7    VDAARIARQKGWL*----------    199
LuxR    CQSISKAILTGAINCPYLK---N*    250
PLR2    QQAIVKCLINNLI*----------    228
CviR    THAIVLGMHLAMTPRELVN---G*    265
PLR1    SAAMALTKELSFS*----------    223
```
