# Supplementary material for: Broad-Host-Range Expression Reveals Native and Host Regulatory Elements That Influence Heterologous Antibiotic Production in Gram-Negative Bacteria
Source: mBio. 2017 Sep 5;8(5):e01291-17. doi: 10.1128/mBio.01291-17 (PMC5587914; doi:10.1128/mBio.01291-17)
Supplement: FIG S7 [file mbo004173462sf7.pdf]

A

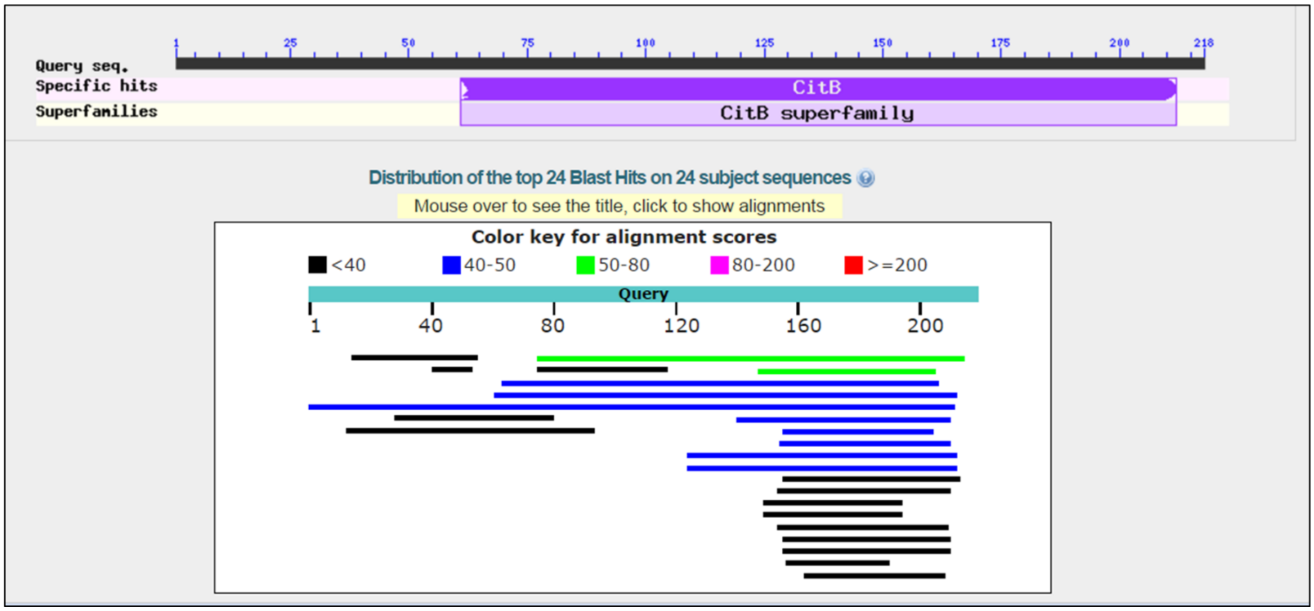

	Gene name	Locus Tag	Score	Coverage (%)	E value	Identity (%)
1	csgD	b1040	68.9	63	3e-15	29
2	yhjB	b3520	50.8	26	1e-08	42
3	uhpA	b3669	49.3	65	3e-08	26
4	narL	b1221	49.3	69	4e-08	29
5	fimZ	b0535	48.5	96	7e-08	22
6	uvrY	b1914	46.2	32	5e-07	35
7	narP	b2193	43.9	22	3e-06	43
8	N/A	EO53_03510	42.7	25	4e-06	41
9	malT	b3418	41.6	40	3e-05	27
10	evgA	b2369	39.3	26	9e-05	33
	sdiA	b1916	33.5	26	0.008	32

	Gene name	Locus Tag	Score	Coverage (%)	E value	Identity (%)	Identical protein?
1	csgD	B21_01044	70.5	63	7e-16	30	N
2	yhjB	B21_03321	50.8	26	8e-09	42	Y
3	uhpA	B21_03495	49.3	65	2e-08	26	Y
4	narL	B21_01209	49.3	69	3e-08	29	Y
5	fimZ	B21_00490	48.5	96	6e-08	22	N
6	N/A	B21_RS09895	46.6	32	2e-07	35	N
7	uvrY	ECD_01879	46.2	32	2e-07	35	N
8	narP	B21_02079	43.9	22	2e-06	43	N
9	malT	B21_03222	41.6	40	2e-05	27	N
10	evgA	B21_02240	39.3	26	7e-05	33	Y

B

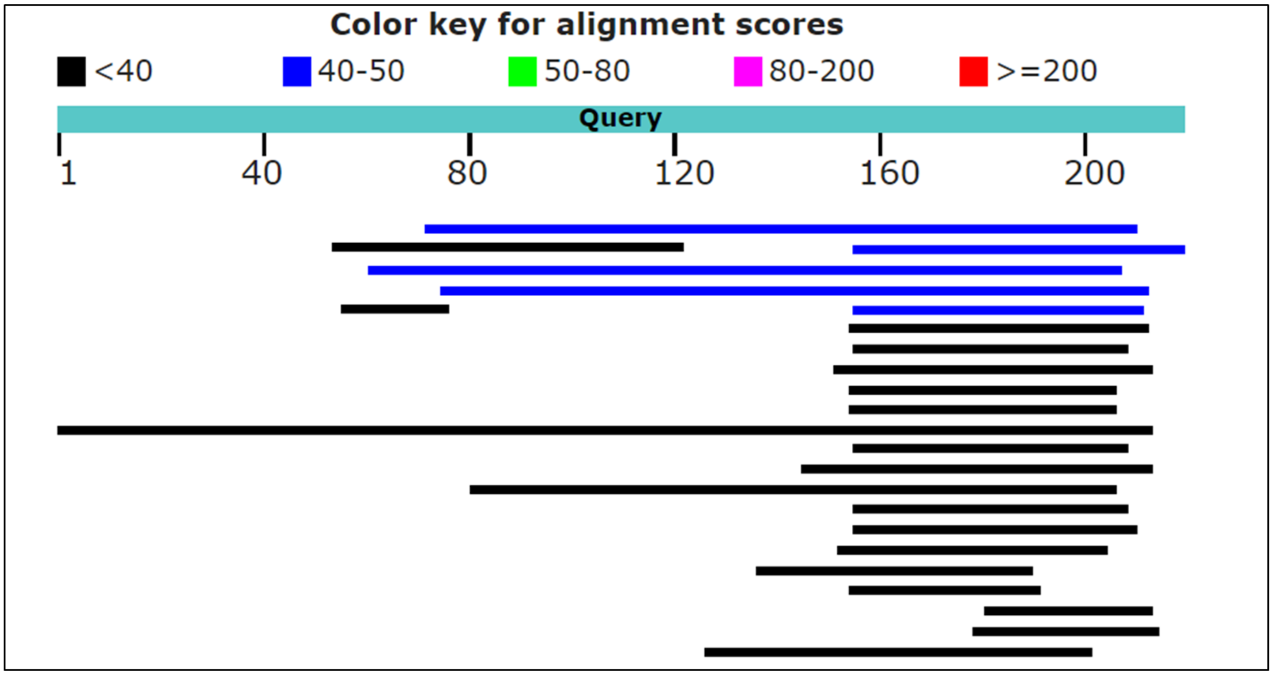

Name	Locus Tag	Score	Coverage (%)	E value	Identity (%)
PPR1	PP_2126	47.4	63	2e-07	25
PPR2	PP_2665	44.3	29	2e-06	35
PPR3	PP_2672	43.1	66	6e-06	23
PPR4	PP_1635	42.4	62	9e-06	21
PPR5	PP_0767	41.6	25	3e-05	38
PPR6	PP_1090	38.9	26	1e-04	31
PPR7	PP_3847	39.3	24	1e-04	42
PPR8	PP_3412	37.4	28	5e-04	29
PPR9	PP_5241	37	23	9e-04	35
PPR10	PP_0574	36.6	23	1e-03	37
PPR11	PP_2101	36.2	97	1e-03	19
PPR12	PP_2587	36.6	24	1e-03	36
PPR13	PP_0410	36.2	31	1e-03	29
PPR14	PP_4099	35.8	57	1e-03	26
PPR15	PP_3717	35.8	24	2e-03	32
PPRX	PP_4647	28.9	22	N/A	29

C

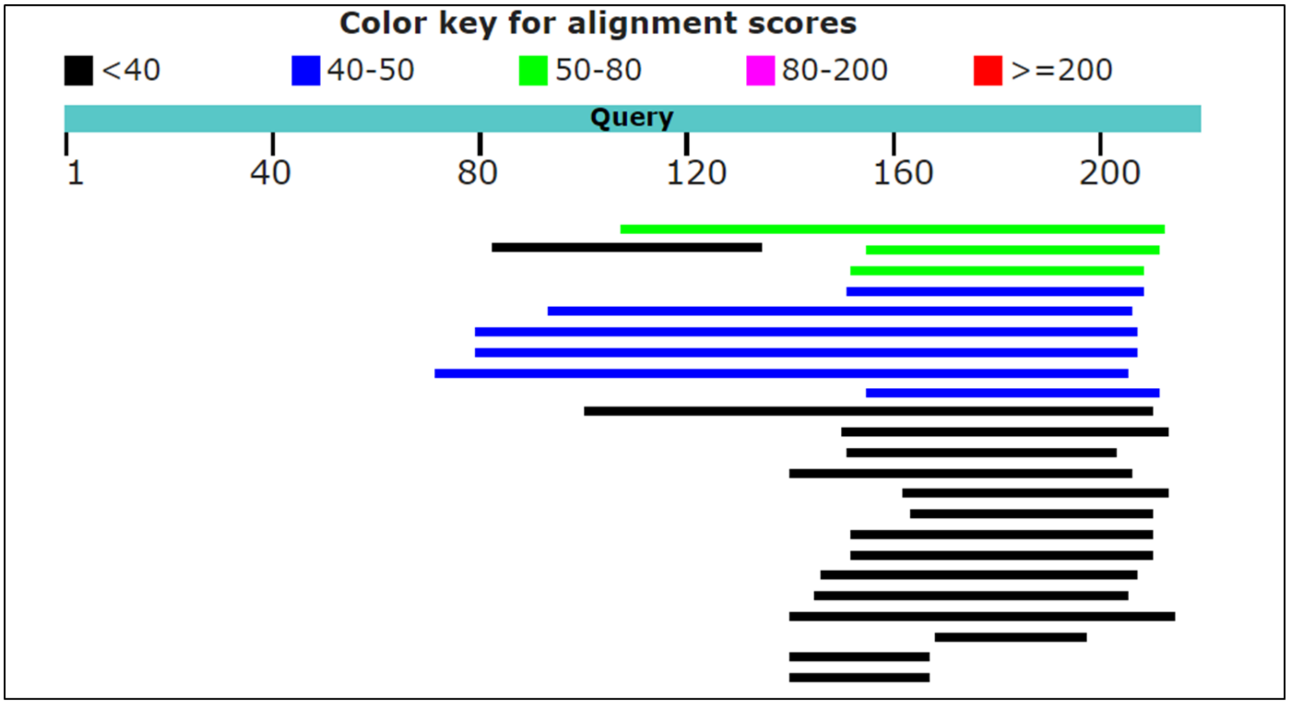

	Locus Tag	Score	Coverage (%)	E value	Identity (%)
**ATR1**	**X971_4629**	**53.9**	**47**	**3e-09**	**27**
**ATR2**	**X971_3376**	**52.4**	**25**	**9e-09**	**45**
**ATR3**	**X971_2241**	**50.8**	**25**	**1e-08**	**45**
ATR4	X971_3295	49.7	26	4e-08	44
ATR5	X971_2958	47.4	51	2e-07	29
ATR6	X971_2949	47	58	3e-07	28
ATR7	X971_0445	42	61	1e-05	23
ATR8	X971_4153	40	25	5e-05	39
ATR9	X971_0488	39.7	50	9e-05	30
ATR10	X971_0487	39.7	28	1e-04	32
ATR11	X971_2373	36.2	23	1e-03	35
ATR12	X971_4763	35.4	30	3e-03	35
ATR13	X971_2720	32.3	23	4e-03	31
**ATRX**	**X971_0678**	**30.8**	**26**	**N/A**	**31**
