# Supplementary material for: Broad-Host-Range Expression Reveals Native and Host Regulatory Elements That Influence Heterologous Antibiotic Production in Gram-Negative Bacteria
Source: mBio. 2017 Sep 5;8(5):e01291-17. doi: 10.1128/mBio.01291-17 (PMC5587914; doi:10.1128/mBio.01291-17)
Supplement: FIG S8 [file mbo004173462sf8.pdf]

```
ATR1      MDDSVNIPKINRLVLQNLIAGLSDGLILLENDGTIAWANKAALQMHRIEAMPELGEDAAA      60
LuxR      ------------------------------------------------------------      0
CviR      ------------------------------------------------------------      0
ATR3      ------------------------------------------------------------      0


ATR1      YRRNFTLRYRNNHLLDEGQYPLERLLAGETFEDVTVEISPSGDEAECWVHTVRGLVLEDA      120
LuxR      ------------------------------------------------------------      0
CviR      ------------------------------------------------------------      0
ATR3      ------------------------------------------------------------      0


ATR1      GAKPDVLVLIIRDETPRFEAEARFESAFNANPAPGLICRLEDKRFIRVNQGFLEMTGFSR      180
LuxR      ------------------------------------------------------------      0
CviR      ------------------------------------------------------------      0
ATR3      ------------------------------------------------------------      0


ATR1      EEIIGISVEELGLFSECDTGEDALKKLEDGRLIRQREALIPIPGGDRLV-----------      229
LuxR      ------------------------------------MNIKNINANEKIIDKIKTCNN---      21
CviR      -----------------------------------MVTSKPINA--R---PLPAGLTASQ      20
ATR3      ---------------------------------------MPKTE--R---QARLCRDLSA      16
                                                        :

ATR1      ------IVAGETIAVAEEPCMLFTFADLDGRRKAQNALRQSEERFFKSFRLSPVPAAISR      283
LuxR      NKDINQCLSEIAKI---IHCEYYLFA----------------I---------IYPHSIIK      53
CviR      QWTLLEWIHMAGHIETEGELKAFLDNILSQAPSDRIILVLGRL---------NNQNQIQR      71
ATR3      AIDRPQWLAALQQV-----MQAFSYSY-------VTLLKLPSI---------RNA---YA      52
                 :              :

ATR1      LDDFVLMEVN----------DAFLVLCGRNEAEVVGKTASELRLWEDAGARRDLEKRLKD      333
LuxR      PDVSIIDNYPEKWR-KYYDDAGLLEYDPVVD---YSKSHHSPINWNVFEKKT--------      101
CviR      MEKVLNVSYPSDWL-NQYSQENFAQHDPIM----RIHLGQGPVIWEERFSRA--------      118
ATR3      LPIVVESSLP-VWVVNAMTRDGELADCPVIK---RGASSMMPQYWSLDDKDI--------      100
              :  .                                    *.

ATR1      NIPIRDENMRMNLSGGGLAECIVSAERAEINDQLCVIWAIQDVTERRRTENELIEAIESV      393
LuxR      -IKKESPNVIKEAQESGLITGFSFPIHTASNGFGMLSFAHSDK--DIYTDSLFLHAS---      155
CviR      -KGSEEKRFIAEASSNGMGSGITFSAASDRNNVGSILSIGGKE--PGRNAALV-------      168
ATR3      -NSGSLLEVSTLLRGMGITSGLLVPVNGMDG-NRHLMNFAGDC--DVLSQGSL-------      149
                 ..       *:   :        .    :     .      .   .

ATR1      MTDTSWFSRTV---VERLAGLRQNSRGTTSSASLKDLTEREEQILSLICDGCSDKEMSDR      450
LuxR      -TNVPLMLPSLVDNYQKIN--------TTRKKSDSILTKREKECLAWASEGKSTWDISKI      206
CviR      -AMLNCLTPHLHQAAVRIAN------LPPASPSNMPLSQREYDIFHWMSRGKTNWEIATI      221
ATR3      -NELCMIALHALEAYDRLC--------RAGSKLPSPLTKRELDVVRWTAQGKTSVEIAEL      200
                :         ::            .     *::** : .   . * :  :::

ATR1      LNLSKHTIRNHIASLYGKIGVNRRTAAVIWARERGFTGHREK*--     492
LuxR      LGCSERTVTFHLTNTQMKLNTTNRCQSISKAILTGAINCPYLKN*     250
CviR      LNISERTVKFHVANVIRKLNANNRTHAIVLGMHLAMTPRELVNG*     265
ATR3      LSISEHTVNTYMNNAIRKLDCVNRAQLVAKTIRLGLIS*------     238
          *. *::*:  :: .   *:.  .*   :      .
```
