# Supplementary material for: Broad-Host-Range Expression Reveals Native and Host Regulatory Elements That Influence Heterologous Antibiotic Production in Gram-Negative Bacteria
Source: mBio. 2017 Sep 5;8(5):e01291-17. doi: 10.1128/mBio.01291-17 (PMC5587914; doi:10.1128/mBio.01291-17)
Supplement: TABLE S1 [file mbo004173462st1.pdf]

Name	Sequence
pCAP05-1_5121F	**AGCTCCAATTCGCCCTATAG**ATCACGAGCAATAAACCCGC
pCAP05-1_5121R	**CTGGAAAGCGGGCAGTGAGC**GGCCTTTTGCTCACATGTTC
pCAP05-2_3943F	**GAACATGTGAGCAAAAGGCC**GCTCACTGCCCGCTTTCCAG
pCAP05-2_3943R	**CCTATTTGTTTATTTTTCTA**ACGATTGATGGCGGTCCTGG
pCAP05-3_2024F	**CCAGGACCGCCATCAATCGT**TAGAAAAATAAACAAATAGG
pCAP05-3_2024R	**GGGTTAATTCCGAGCTTGGC**TATTTCACACCGCATATGAT
pCAP05-4_1639F	**ATCATATGCGGTGTGAAATA**GCCAAGCTCGGAATTAACCC
pCAP05-4_1639R	**GCGGGTTTATTGCTCGTGAT**CTATAGGGCGAATTGGAGCT
vio2ta16-1_3121-40F	**CTCGGTTTGACGCCTCCCATGGTATAAATAGTGGCTCGAG**CCTAAACTAACCGCCATGTG
vio2ta16-1_3121-134R	CTGTAGTAGCTGCGTATGTCCAGTGACTGT
vio2ta16-2_3058-134F	TGCTCAGAGCGGCACACTTG
vio2ta16-2_3058-108R	TCTGGCGGGTAATGATCTGG
vio2ta16-3b_3027-482F	GCGGTTTGCCTTCCTTATACGGGTCAAGTG
vio2ta16-3b_3027-40R	**AGTAGCAGCACGTTCCTTATATGTAGCTTTCGACATCGAT**CCGCGGGTTTCCTAAATTAC
vio2ta16check_402F	CTCGGGAGATGCCAGAAGTC
vio2ta16check_402R	GCTCGCCCAATGCCATAAGG
pET28a-vioE-F	**TGAAATTTTTCAACCGACTCCATTAAAACAACCTGAGTAA**GCACTCGAGCACCACCACCA
pET28a-vioA-R	**CCGCTCCGATGATTGAGATGGTGTTTTCTCTAACCGTCAT**GTATATCTCCTTCTTAAAGT
PLR1(NcoI)_669F	**ACCTGCCCATGGGC**TTTTTGGGCTGCACAAAAGA
PLR1(HindIII)_669R	**GGCCGCAAGCTT**TTAGCTAAAGCTCAGCTCTTTG
PLR2(NcoI)_684F	**ACCTGCCCATGGGC**AAACCTACCGAACAAGATAG
PLR2(HindIII)_684R	**GGCCGCAAGCTT**CTAAATAAGGTTGTTTATTAAACACT
PLR3(NcoI)_654F	**ACCTGCCCATGGGC**AATCAGTTTTTAATTGCGGATGA
PLR3(HindIII)_654R	**GGCCGCAAGCTT**CTACTTTGCCGCCTCGATT
PLR4(NcoI)_657F	**ACCTGCCCATGGGC**AGCAGTACTGCATTTTTATTG
PLR4(HindIII)_657R	**GGCCGCAAGCTT**TTAACGTATCTCGTCAGGGA
PLR5(NdeI)_642F	**CGGAATTCCATATG**GGTAACGGAAAAGAAAATAG
PLR5(XhoI)_642R	**AACCGCTCGAG**CTACACAACTAGCCCTTCTT
PLR6(NcoI)_717F	**ACCTGCCCATGGGC**AAAGCGATTCAAACTAGCAA
PLR6(HindIII)_717R	**GGCCGCAAGCTT**TTAATGACGCTGTATTGAGA
PLR7(NdeI)_600F	**CGGAATTCCATATG**AAAATTTTAGTTGTAGAAGATCAA

PLR7(XhoI)_600R	**AACCGCTCGAG**TTACAGCCAACCCTTTTGTC
qRT-cysG_126F	TCCACAGTTCACCGCATGGG
qRT-cysG_126R	CGCTGGTTAAGCGCGTCATC
qRT-vioA_140F	CTGACAATGCTGGCTATGAG
qRT-vioA_140R	CGCAGTATCAGCTTCTAGAC
csgD(NdeI)_651F	**CGGAATTCCATATG**TTTAATGAAGTCCATAGTATTCATG
csgD(XhoI)_651R	**AACCGCTCGAG**TTATCGCCTGAGGTTATCGT
yhjB(NcoI)_603F	**ACCTGCCCATGGGC**CAAATAGTCATGTTTGACAG
yhjB(HindIII)_603R	**GGCCGCAAGCTT**TCAGGAGGAGATATTTAACA
uhpA(NcoI)_591F	**ACCTGCCCATGGGC**ATCACCGTTGCCCTTATAGA
uhpA(HindIII)_591R	**GGCCGCAAGCTT**TCACCAGCCATCAAACATGC
sdiA(NcoI)_723F	**ACCTGCCCATGGGC**CAGGATAAGGATTTTTTCAGCT
sdiA(HindIII)_723R	**GGCCGCAAGCTT**TCAAATTAAGCCAGTAGCGG
PPR1(NdeI)_660F	**CGGAATTCCATATG**ACCTGTAGACTACTGCT
PPR1(XhoI)_660R	**AACCGCTCGAG**TCAGTCATCCAGGCTGATGA
PPR2(NcoI)_666F	**ACCTGCCCATGGGC**TACAAAATTCTGATTGCCGAC
PPR2(HindIII)_666R	**GGCCGCAAGCTT**TCAACGGCGCAGATAATCA
PPR5(NdeI)_2718F	**CGGAATTCCATATG**ACAGATCTGTCCCGTACCCA
PPR5(XhoI)_2718R	**AACCGCTCGAG**TCACGCCAGCAACCCCAGG
PPRX(NcoI)_708F	**ACCTGCCCATGGGC**CTTCACTGGAAACCCGA
PPRX(HindIII)_708R	**GGCCGCAAGCTT**TCAGATCCAGCCACGCAAGG
ATR1(NdeI)_1479F	**CGGAATTCCATATG**GACGACAGCGTAAACAT
ATR1(XhoI)_1479R	**AACCGCTCGAG**TTATTTTTCCCTATGGCCGG
ATR2(NdeI)_930F	**CGGAATTCCATATG**AGCGGGCAAGGCGCAG
ATR2(XhoI)_930R	**AACCGCTCGAG**TCACCCCTGATGCAAAATATGCGCG
ATR3(NcoI)_717F	**ACCTGCCCATGGGC**CCGAAGACGGAGCGCCAGG
ATR3(HindIII)_717R	**GGCCGCAAGCTT**TCAGCTTATCAGGCCAAGGCG
ATRX(NcoI)_756F	**ACCTACCATGGGC**AAGAACCGCTTCATGCA
ATRX(HindIII)_756R	**GGCCGCAAGCTT**CTATTTAATCAGACCGACCC

**Name**	**Description**	**Reference**
pARS-VN	Plasmid used to amplify selective, counter-selective, and maintenance elements for yeast TAR cloning in construction of pCAP05	(1)
pRK442(H)	Plasmid used to amplify broad-host-range origin of replication and maintenance elements for Gram-negative bacteria in construction of pCAP05	(2)
pCAP05	TAR cloning and broad-host-range heterologous expression vector; CEN6_ARS4, *HIS3*, p*AHD1*, *URA3*, *oriV*, *traJ*, *oriT*, *tetR*, *tetA*, *trfA*, *incC*, *korA*	This study
pCAP05-vio2ta16	pCAP05 containing violacein gene cluster from *P. luteoviolcea* 2ta16; Tet^R	This study
pET28a-vio2ta16	pET28a containing violacein gene cluster from *P. luteoviolcea* 2ta16 under control of T7 promoter; Kan^R	This study
pACYCDuet-1	Commercial expression vector used for co-expression of LuxR homologs; Cm^R	Novagen

1. Noskov VN, Kouprina N, Leem SH, Ouspenski I, Barrett JC, Larionov V. 2003. A general cloning system to selectively isolate any eukaryotic or prokaryotic genomic region in yeast. BMC Genomics 4:16.
2. Scott HN, Laible PD, Hanson DK. 2003. Sequences of versatile broad-host-range vectors of the RK2 family. Plasmid 50:74-9.
